# Therapeutic benefits of phosphodiesterase 4B inhibition after traumatic brain injury

**DOI:** 10.1371/journal.pone.0178013

**Published:** 2017-05-19

**Authors:** Nicole M. Wilson, Mark E. Gurney, W. Dalton Dietrich, Coleen M. Atkins

**Affiliations:** 1 Department of Neurological Surgery, The Miami Project to Cure Paralysis, University of Miami Miller School of Medicine, Miami, Florida, United States of America; 2 Tetra Discovery Partners, Grand Rapids, Michigan, United States of America; University of South Florida, UNITED STATES

## Abstract

Traumatic brain injury (TBI) initiates a deleterious inflammatory response that exacerbates pathology and worsens outcome. This inflammatory response is partially mediated by a reduction in cAMP and a concomitant upregulation of cAMP-hydrolyzing phosphodiesterases (PDEs) acutely after TBI. The PDE4B subfamily, specifically PDE4B2, has been found to regulate cAMP in inflammatory cells, such as neutrophils, macrophages and microglia. To determine if PDE4B regulates inflammation and subsequent pathology after TBI, adult male Sprague Dawley rats received sham surgery or moderate parasagittal fluid-percussion brain injury (2 ± 0.2 atm) and were then treated with a PDE4B - selective inhibitor, A33, or vehicle for up to 3 days post-surgery. Treatment with A33 reduced markers of microglial activation and neutrophil infiltration at 3 and 24 hrs after TBI, respectively. A33 treatment also reduced cortical contusion volume at 3 days post-injury. To determine whether this treatment paradigm attenuated TBI-induced behavioral deficits, animals were evaluated over a period of 6 weeks after surgery for forelimb placement asymmetry, contextual fear conditioning, water maze performance and spatial working memory. A33 treatment significantly improved contextual fear conditioning and water maze retention at 24 hrs post-training. However, this treatment did not rescue sensorimotor or working memory deficits. At 2 months after surgery, atrophy and neuronal loss were measured. A33 treatment significantly reduced neuronal loss in the pericontusional cortex and hippocampal CA3 region. This treatment paradigm also reduced cortical, but not hippocampal, atrophy. Overall, these results suggest that acute PDE4B inhibition may be a viable treatment to reduce inflammation, pathology and memory deficits after TBI.

## Introduction

Every year approximately 1.7 million people suffer a traumatic brain injury (TBI) in the United States [1]. This is a serious clinical problem that results in an estimated 3–5.3 million people living with lasting TBI-related disabilities, at a total cost of approximately $60 billion dollars annually [1–3]. A majority of TBI survivors go on to develop cognitive impairments, often resulting in reduced quality of life and increased economic burden for the individual and their family [4–8]. Treatments aimed at reducing inflammation and, consequently, the extent of damage after TBI are a potential strategy for attenuating these cognitive impairments. In experimental models of CNS injury, elevating cAMP levels through inhibition of cAMP-hydrolyzing phosphodiesterases (PDEs) is an effective strategy for reducing inflammation and improving outcome [9]. Development of an inhibitor to target selective PDE isoforms involved in inflammation after TBI would greatly facilitate clinical development.

The anti-inflammatory benefits of increasing cAMP levels are well documented in experimental models of TBI, spinal cord injury (SCI), cerebral ischemia and multiple sclerosis (MS) [10–13]. Of the 11 identified PDE families, PDE4, PDE7 and PDE8 are specific for cAMP [9, 14]. In the context of inflammation and CNS injury, PDE4 is one of the more extensively studied cAMP-specific PDEs and is a major cAMP-hydrolyzing enzyme in the brain [15–18]. The PDE4 superfamily consists of 4 subfamilies: PDE4A, 4B, 4C and 4D. Each subfamily is encoded by a single gene, and there are multiple isoforms within each subfamily [14, 19]. Pan-PDE4 inhibitors, such as rolipram, have been used to reduce tumor necrosis factor (TNF) levels and neutrophil accumulation in models of systemic inflammation and CNS injury, such as SCI, cerebral ischemia and TBI [10, 12, 13, 20]. Previously, we reported that pre-injury treatment with rolipram rescued cAMP signaling deficits and reduced inflammation after TBI, as measured by a decrease in TNF and interleukin-1β (IL-1β) levels [12]. When administered after TBI, rolipram reproduced the anti-inflammatory benefits observed with pre-injury treatment, but also resulted in increased hemorrhage [21, 22]. These results suggest that the anti-inflammatory benefits of acute pan-PDE4 inhibition are overshadowed by vascular perturbations after TBI [21, 22]. Altogether, the hemorrhagic and emetic effects observed with pan-PDE4 inhibitors underscores the need for more refined approaches for targeting PDE4 subfamilies to reduce inflammation and improve recovery after TBI [23, 24].

The development of PDE4 subfamily knockout mice has further clarified the functions of each of these subfamilies. One of the major findings is the role of the PDE4B subfamily in regulating inflammatory signaling. Specifically, PDE4B has been shown to regulate TNF production and neutrophil recruitment [25, 26]. After TBI, neutrophils are the first immune cell population to infiltrate the injured brain [27]. Neutrophils are found in the brain within a few hours after trauma, and peak accumulation occurs around 24 hrs after injury [28, 29]. Preclinical studies have demonstrated that neutrophil accumulation is associated with poor histopathological and behavioral outcome after TBI, and treatments aimed at reducing neutrophil accumulation attenuate these TBI-induced pathologies [30–33]. These studies suggest that treatments for reducing neutrophil accumulation, such as PDE4B inhibition, are potential therapeutics for attenuating inflammation and improving outcome after TBI.

In addition to regulating neutrophil recruitment, several studies have demonstrated that augmenting cAMP signaling leads to an upregulation of anti-inflammatory markers, such as Arginase 1 (Arg1), in macrophages and microglia [34–38]. Arg1 and inducible nitric oxide synthase (iNOS) are competitive enzymes, and the dichotomous relationship between iNOS and Arg1 in regulating L-arginine metabolism and inflammation has led to the use of these enzymes as pro—and anti-inflammatory markers, respectively [39–41]. However, whether the PDE4B subfamily is responsible for modulating cAMP signaling in iNOS or Arg1-producing pathways in inflammatory cells after TBI is unknown.

The PDE4B subfamily consists of 5 known isoforms, PDE4B1–5. In the context of inflammation, the PDE4B2 isoform is of particular importance due to its regulation by inflammatory stimuli such as lipopolysaccharide (LPS) and TNF [15, 42, 43]. Furthermore, increased expression of PDE4B2 has been associated with a pro-inflammatory phenotype in macrophages, microglia and neutrophils [15, 42–44]. Increased PDE4B2 expression has also been reported in experimental models of SCI, MS and TBI [15, 45, 46]. Altogether, these findings suggest a potential role for PDE4B2 in regulating the inflammatory response in multiple CNS injury and disease models, including TBI.

The PDE4B-specific inhibitor, A33, has made it possible to study the therapeutic benefits of targeting PDE4B in a variety of neurological conditions [47, 48]. A33 has a half-life of approximately 3.8–4.5 hrs in the mouse brain, and is over 50-fold more selective for PDE4B (IC_50_ = 27 nM) over PDE4D (IC_50_ = 1569 nM) and other PDEs (IC_50_ > 10μM) [49, 50]. This selectivity is due to a single amino acid polymorphism in the C-terminus of PDE4B, termed CR3 (Control Region 3) [49, 51]. CR3 is conserved in all PDE4B isoforms and co-crystal structure studies have revealed that A33 inhibits PDE4B by promoting a closed conformation [49, 51]. The A33-mediated inactive conformation is due to CR3 closing over the active site of PDE4B and preventing access to cAMP [49, 51]. Overall, the improved specificity of A33 over available pan-PDE4 inhibitors that access the CNS, such as rolipram, makes it an important reference compound for evaluating the anti-inflammatory effects of PDE4B inhibition.

The connection between inflammation and cAMP signaling has been extensively studied in multiple models of CNS injury and disease [11, 13, 15, 52]. However, lack of subtype-selective PDE4 inhibitors has hampered the development of this therapeutic approach for TBI [21, 22]. In this study, we characterized the effect of a PDE4B-selective inhibitor, A33, on inflammation, pathology and behavioral deficits after TBI. We report that treatment with A33 reduced neutrophil accumulation, microglia activation, cortical contusion volume, memory deficits, neuronal loss and cortical atrophy after TBI. These results support the use of PDE4B-selective inhibitors as a treatment for TBI.

## Materials and methods

### Surgery

All experimental procedures were in compliance with the NIH Guide for the Care and Use of Laboratory Animals and approved by the University of Miami Animal Care and Use Committee (IACUC protocol number: 17–004). Prior to surgery, animals were randomized to receive sham or TBI surgery, and treatment with A33 or vehicle. To determine the minimum number of animals needed for each experiment, a power analysis was performed to detect a 20% difference between treatment groups with power set at 80% and significance at 0.05. A minimum n value of 3 for biochemical studies and 10 for behavioral measures was determined. Adult male Sprague Dawley rats (2–3 months old, Charles River Laboratories) were anesthetized (3% isoflurane, 70% N_2_O, 30% O_2_) and received a 4.8 mm diameter craniotomy at 3.8 mm posterior to bregma and 2.5 mm lateral to midline over the right parietal cortex. A plastic luer lock adapter was fastened at the craniotomy site with cyanoacrylate and dental cement. Animals were fasted overnight (12–16 hrs) with water *ad libitum*. Animals were re-anesthetized (5 min induction with 3% isoflurane, 70% N_2_O, 30% O_2_, maintenance with 1% isoflurane, 70% N_2_O, 30% O_2_), intubated and mechanically ventilated (Stoelting) and given rocuronium (10 mg/kg, i.a.) and penicillin G (20,000 IU/kg, i.m.). Head and body temperature were maintained at normothermic temperatures (37 ± 0.5°C) using rectal and temporalis muscle thermistors connected to feedback-regulated heating lamps. Physiological parameters (blood pO_2_, pCO_2_ and pH, mean arterial blood pressure) were monitored via a tail artery catheter and maintained at normal levels. Brain trauma was produced with a fluid-pulse (14–16 ms duration, 2.0 ± 0.2 atm) at the craniotomy site. Sham-operated animals received all surgical procedures identical to the TBI animals with the exception of the fluid-pulse. At the end of the surgery, animals received buprenorphine (0.01 mg/kg, s.c.) to minimize pain and distress. Criteria for exclusion from the study were: mortality, >15% loss of body weight, non-resolving infection at the surgical site, inability to feed or drink, motor paralysis, listlessness, self-mutilation, excessive grooming leading to loss of dermal layers, spontaneous vocalization when touched or poor grooming habits. Attrition rate was 3.5% for sham animals (1 animal due to mortality, 1 due to infection) and 7% for TBI animals (4 animals due to mortality after surgery).

### A33 synthesis

Compound A33 (2-(4-([2-(5-chlorothiophen-2-yl)-5-ethyl-6-methylpyrimidin-4-yl]amino)phenyl)acetic acid; CAS 915082-52-9) was synthesized as described previously [48].

### Flow cytometry

For flow cytometry experiments, animals received either vehicle (5% DMSO in saline, 6 ml/kg, i.p.) or A33 (0.3 mg/kg, 6 ml/kg, i.p.) at 30 min after TBI. For the 24 hr endpoint, animals received a second dose of either vehicle or A33 at 5 hrs post-surgery. At 3 or 24 hrs post-surgery, animals were deeply anesthetized (3% isoflurane, 70% N_2_O, 30% O_2_, 5 min) and transcardially perfused with PBS (4°C), pH 7.4, (75 mL) for 6 minutes. The ipsilateral parietal cortex was dissected on ice. Tissue was mechanically dissociated into a single-cell suspension and cells were labeled for surface markers CD45 Alexa 647 (202212, 1.25 μg/ml, BioLegend) and CD11b v450 (53-4321-80, 1 μg/ml, eBioscience). The surface markers CD45 and CD11b were used to distinguish between CD45^low^ microglia (CD45^low^, CD11b^+^) and CD45^high^ infiltrating myeloid-lineage cells (CD45^high^, CD11b^+^). Neutrophils are a subset of infiltrating myeloid-lineage cells and were labeled with RP-1 PE (550002, 20 μg/ml, BD Bioscience) to distinguish them from other CD45^high^ cells. Dead cells were excluded from analysis by labeling with LIVE/DEAD Fixable Near-IR dead cell stain (L10119, 1 μl/ml, Life Technologies). Cells were fixed and permeabilized with BD Cytofix/Cytoperm Fixation/Permeabilization kit (554714, BD Biosciences). For intracellular labeling, the cells were probed for either PDE4B2 (ABS181, 2 μg/ml, EMD Millipore), or iNOS Alexa 488 (53–5920, 5 μg/ml, eBioscience) and Arg1 PE (IC5868P, 7.5 μg/ml, R&D Systems). PDE4B2 staining was detected with PE-conjugated secondary antibody (12-4739-81, 10 μg/ml, eBioscience). Antibody isotype controls provided by manufacturers were used to establish gates during analysis. Flow cytometry data was acquired on a BD LSR II flow cytometer (BD Biosciences). Data collection was performed using BD FACSDiva 8.0.1 (BD Biosciences) and analyzed with Kaluza 1.2 software (Beckman Coulter).

### Brain and plasma A33 levels

At 30 min and 5 hrs post-surgery, animals received A33 (0.3 mg/kg, 6 ml/kg, i.p.). At 6 hrs post-surgery, animals were anesthetized (3% isoflurane, 70% N_2_O, 30% O_2_, 5 min) and decapitated. The ipsilateral and contralateral parietal cortex and hippocampus were dissected, snap frozen in liquid nitrogen and stored at -80°C. The cortical and hippocampal tissue from each side were combined for analysis. Trunk blood was collected after decapitation, diluted with 500 mM K^+^—EDTA (pH 8.0) and centrifuged at 3000 x g (10 min, 4°C). Plasma was removed and stored at -80°C. Protein was precipitated with 0.1% formic acid and A33 levels were quantified by liquid chromatography-tandem mass spectrometry as previously described [50].

### cAMP ELISA

Animals received either A33 (0.3 mg/kg, 6 ml/kg, i.p.), or vehicle (5% DMSO in saline, 6 ml/kg, i.p.) at 30 min and 5 hrs post-surgery. At 6 hrs post-surgery the animals were anesthetized (3% isoflurane, 70% N_2_O, 30% O_2_, 5 min), decapitated and the ipsilateral parietal cortex was dissected at 4°C, snap frozen in liquid nitrogen and stored at -80°C. Samples were assayed in duplicate according to the manufacturer’s protocol using a cAMP ELISA (ADI-900-066, Enzo Life Science), and normalized to total protein using a Coomassie Plus assay (23236, ThermoFisher Scientific).

### Cortical contusion volume

Animals received vehicle (5% DMSO in saline, 6 ml/kg, i.p.) or A33 (0.3 mg/kg, 6 ml/kg, i.p.) at 30 min post-TBI and once daily for 3 days. At 3 days post-surgery, animals were anesthetized (3% isoflurane, 70% N_2_O, 30% O_2_, 5 min), and transcardially perfused with 0.9% isotonic saline (80 mL), followed by 4% paraformaldehyde in 0.1 M phosphate buffer (4°C, 210 mL), pH 7.4. The brains were embedded in paraffin, sectioned (10 μm thick) and stained with hematoxylin and eosin (H&E). Cortical contusion volume was evaluated in serial brain sections (150 μm apart) by contouring the entire contusion at 20x magnification using Neurolucida 10.50.2 (MBF Bioscience) and an Olympus BX51 microscope (Olympus America). The cortical contusion boundaries were defined by edematous tissue, pyknotic cells and an accumulation of red blood cells and infiltrating leukocytes at the border between the parietal cortex and external capsule.

### Behavioral experiments

Animals received sham surgery or moderate parasagittal fluid-percussion brain injury and were then given A33 (0.3 mg/kg, 6 ml/kg, i.p.) or vehicle (saline, 6 ml/kg, i.p.) at 30 min and 5 hrs post-TBI, and once daily for 3 days. From 1–6 weeks post-surgery, animals were then tested serially on the cylinder test (1 week post-surgery), contextual fear conditioning (2 and 6 weeks post-surgery), water maze (3 weeks post-surgery) and spatial working memory (4 weeks post-surgery). At 8 weeks post-surgery, animals were perfused and their brains were evaluated for atrophy and neuronal loss. All behavioral tests and histopathological assessments were conducted by an investigator blinded to treatment groups.

### Cylinder test

Animals were evaluated for spontaneous forelimb placement in a transparent Plexiglas cylinder (20 cm diameter x 30 cm height) for 5 min. The cylinder dimensions encouraged vertical exploration [53]. Animals were first evaluated for baseline behavior in the cylinder test at 1–3 days prior to surgery, and were re-evaluated at 1 week post-surgery. The number of times the right or left forelimb made contact with the wall while the animal was rearing was counted. Asymmetry index was calculated by dividing the number of contralateral (left) forelimb touches by total forelimb touches. For each animal, asymmetry index at 1 week post-surgery was normalized to baseline asymmetry index to account for any pre-operative bias [54].

### Fear conditioning

Animals were habituated to the fear conditioning apparatus (30.5 x 24.1 x 21 cm, Coulbourn Instruments) for 10 min to facilitate contextual fear conditioning [55]. The following day, animals were returned to the apparatus for 3.5 min. After the initial 2 min, a 30 sec tone (75 dB, 2.8 kHz) was delivered that coterminated with a 1 mA foot shock (1 sec duration). Animals remained in the apparatus for 1 min post-shock. The apparatus was cleaned with 70% ethanol between each trial. At 24 hrs and 1 month (6 weeks post-surgery) after training, contextual fear conditioning was evaluated by placing the animals in the apparatus and measuring freezing for 5 min. Contextual fear conditioning comparisons were made between freezing on the training day during the initial 2 min, designated as training, and freezing in the context on the testing days. Video-based analysis was used to quantify freezing behavior (FreezeFrame 3.32, Coulbourn Instruments). Shock threshold was measured at 6 weeks post-surgery after completion of contextual fear conditioning. Animals received a 1 sec foot shock every 30 sec in 0.02 mA increments beginning at 0.1 mA. The minimum shock intensity required to elicit a flinch, jump or vocalization was recorded.

### Water maze

Animals were tested for spatial memory deficits using a water maze. The circular pool (122 cm diameter, 60 cm height) used for the water maze was filled with opaque water (24°C) and surrounded by distinct extra-maze cues. The escape platform (9.3 cm diameter) was submerged 1.5 cm below the surface of the water and remained invariant in location. Animals received four 60 sec acquisition trials per day for 4 days with an intertrial interval of 4–6 min. If the animal was unable to reach the platform within 60 sec, it was guided to the platform and remained on the platform for 10 sec. After 4 training days, a probe trial (90 sec duration) was given with the platform removed. Escape latency, path length, time spent in target quadrant and average velocity were analyzed with EthoVision XT 10 software (Noldus Information Technology).

### Working memory

Using a water maze, animals received 4 paired trials per day for 2 days with a 5 sec delay between trials. The platform and release location remained invariant for each paired trial. Maximum trial duration was 60 sec. If the animal did not reach the platform within 60 sec, it was guided to the platform and remained on the platform for 10 sec. After a 5 sec delay, the animal was released into the water at the previous release location. The escape path length for the location and match trials on day 2 were analyzed using EthoVision XT 10 software (Noldus Information Technology).

### Atrophy

At the completion of behavioral testing, animals were anesthetized (3% isoflurane, 70% N_2_O, 30% O_2_, 5 min) and transcardially perfused with 0.9% isotonic saline (80 mL), followed by 4% paraformaldehyde in 0.1 M phosphate buffer (4°C, 210 mL), pH 7.4. The brains were embedded in paraffin, sectioned (10 μm thick) and stained with H&E plus Luxol fast blue. Slides were digitized at 7200 dpi (3.5 μm/pixel) using Quickscan PathScan Enabler IV (version 3.60.0.12). Serial sections (150 μm apart) were evaluated for atrophy by contouring the ipsilateral and contralateral cortex and hippocampi from -3.3 to -6.8 mm bregma using Neurolucida 11.11.2 software (MBF Bioscience). To account for differences in tissue shrinkage, percent atrophy was determined by calculating the difference between ipsilateral and contralateral volume, and normalizing to the contralateral volume. Representative images were acquired at 20x using an Olympus BX51 microscope (Olympus America) and StereoInvestigator 5.65 software (MBF Bioscience).

### Neuronal counts

Sections were antigen-retrieved using citrate buffer (10 mM citrate, pH 6.0, 80°C) for 20 min, blocked for 1 hr at RT in blocking buffer (PBS containing 3% normal horse serum and 0.4% TX-100), then incubated overnight at 4°C with mouse anti-NeuN antibodies (clone A60, MAB377, 1:500, EMD Millipore) in blocking buffer. Sections were rinsed with PBS and 0.4% TX-100, and then incubated with biotinylated horse anti-mouse antibodies (BA-2001, 1:200, Vector Laboratories) for 90 min at RT in blocking buffer. Sections were rinsed with PBS and 0.4% TX-100 and incubated with VECTASTAIN Elite ABC HRP (PK-6100, Vector Laboratories) for 90 min at RT in PBS and 0.4% TX-100. Sections were then rinsed in PBS and developed for up to 3 minutes at RT in Sodium Acetate (50 mM)—Imidazole Buffer (15 mM), pH 7.2, supplemented with 2.5% Nickel Ammonium Sulfate (w/v), 0.05% diaminobenzene (w/v) and 0.009% H_2_O_2_ (v/v). Sections were processed in parallel for immunohistochemistry. The parietal cortex and CA3 region of the hippocampus were contoured near the epicenter of injury from -5.0 to -5.6 mm bregma at 4x magnification using an Olympus BX51 microscope (Olympus America) and StereoInvestigator 5.65 software (MBF Bioscience). NeuN^+^ cells were counted at 60x magnification (1.42 NA objective) using a 35 x 35 μm counting frame. A 141 x 92 μm sampling grid was used for the CA3 region of the hippocampus and a 200 x 200 μm sampling grid was used for the parietal cortex. For hippocampal CA3 cell counts, Q values ranged from 106–285 and CE^2^/CV^2^ values were 0.0001, 0.0005 and 0.0002 for sham, vehicle- and A33-treated TBI animals, respectively. For cortical cell counts, Q values ranged from 242–697 and CE^2^/CV^2^ values were 0.00001, 0.00039 and 0.00009 for sham, vehicle- and A33-treated TBI animals, respectively. Representative images were acquired at 20x using an Olympus BX51 microscope (Olympus America) and StereoInvestigator 5.65 software (MBF Bioscience).

### Data analysis

Data presented are mean ± SEM. Significance was designated at *p*<0.05. Statistical comparisons were made using GraphPad Prism 6.05 and SigmaPlot 11.0. Plasma A33 levels, flow cytometry and cortical contusion volume were analyzed using an unpaired Student’s t-test. Asymmetry index, cAMP ELISA, shock threshold, time spent in target quadrant, swim velocity, working memory match trials and cortical and hippocampal atrophy results were analyzed using a one-way ANOVA and post-hoc Student-Newman-Keuls. Contextual fear conditioning, water maze escape latency and path length were assessed using a repeated-measures two-way ANOVA (treatment x trial) and post-hoc Student-Newman-Keuls. Brain A33 levels (surgery x ipsi/contra), working memory path length (treatment x trial), and cortical and hippocampal CA3 neuronal counts (treatment x ipsi/contra) were assessed using a two-way ANOVA and post-hoc Student-Newman-Keuls.

## Results

### PDE4B2 is expressed in inflammatory cells acutely after TBI

**Fig 1:**
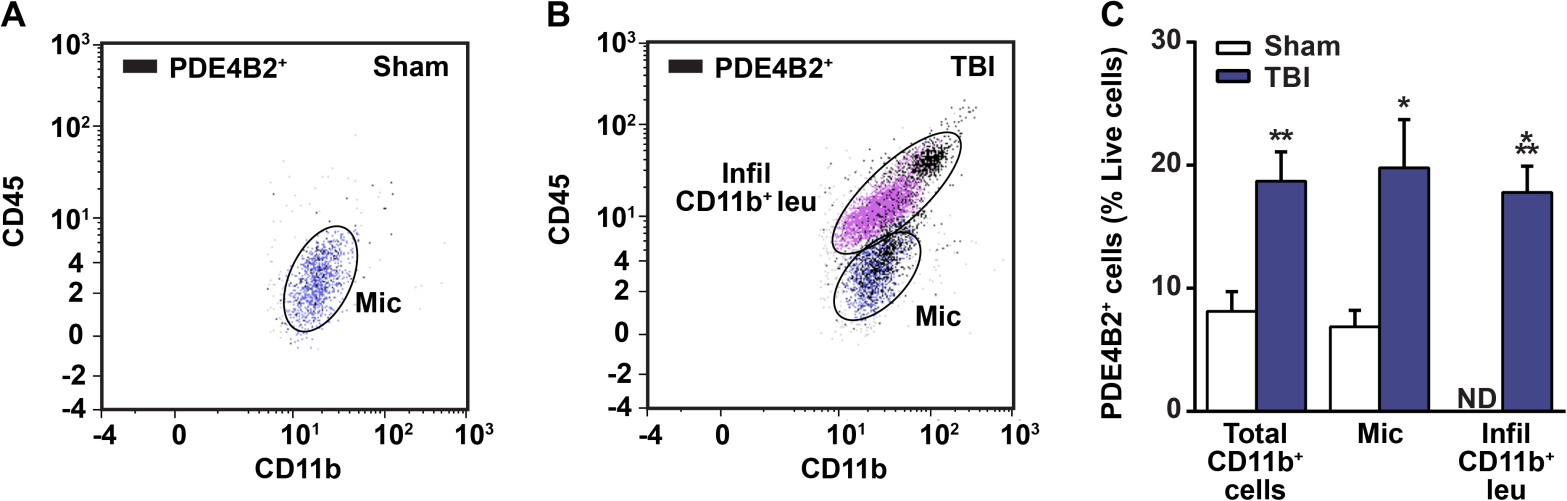
PDE4B2 is expressed in microglia and infiltrating myeloid-lineage cells in the injured cortex at 24 hrs post-TBI. Representative scatter plots from the ipsilateral parietal cortex of **(A)** sham and **(B)** brain-injured animals. **(C)** Quantification of PDE4B2^+^ inflammatory cells in the cortex at 24 hrs post-surgery. Mean ± SEM, *n* = 8-10/group, *p<0.05, **p<0.01, ***p<0.001 vs. Sham, Student’s unpaired t-test. ND = not detectable.

PDE4B2 has been implicated in the acute inflammatory response in multiple CNS injury and disease models [15, 46]. However, whether PDE4B2 has a role in the acute inflammatory response after TBI was unknown. To evaluate this, we used flow cytometry to determine whether PDE4B2 expression was increased in microglia (CD45^low^, CD11b^+^) and infiltrating myeloid-lineage cells (CD45^high^, CD11b^+^) at 24 hrs after TBI. This time point was selected because PDE4B2 is elevated in the injured cortex at 24 hrs after TBI, and it also corresponds to the peak of neutrophil infiltration [27, 30, 45]. At 24 hrs post-surgery, there were few PDE4B2^+^/CD11b^+^ cells in the ipsilateral parietal cortex of sham animals (**Fig 1A**), and this nearly doubled in TBI animals (**Fig 1B and 1C**). The increase in PDE4B2^+^/CD11b^+^ cells after TBI was due to both an increase in PDE4B2-expressing microglia and the infiltration of PDE4B2^+^ myeloid-lineage cells (**Fig 1B and 1C**). These results suggest that the previously reported increase in PDE4B2 expression after TBI by western blotting may be due, in part, to an increase in both PDE4B2-expressing microglia and myeloid-lineage cells [45].

### Levels of A33 in the brain and plasma

**Fig 2:**
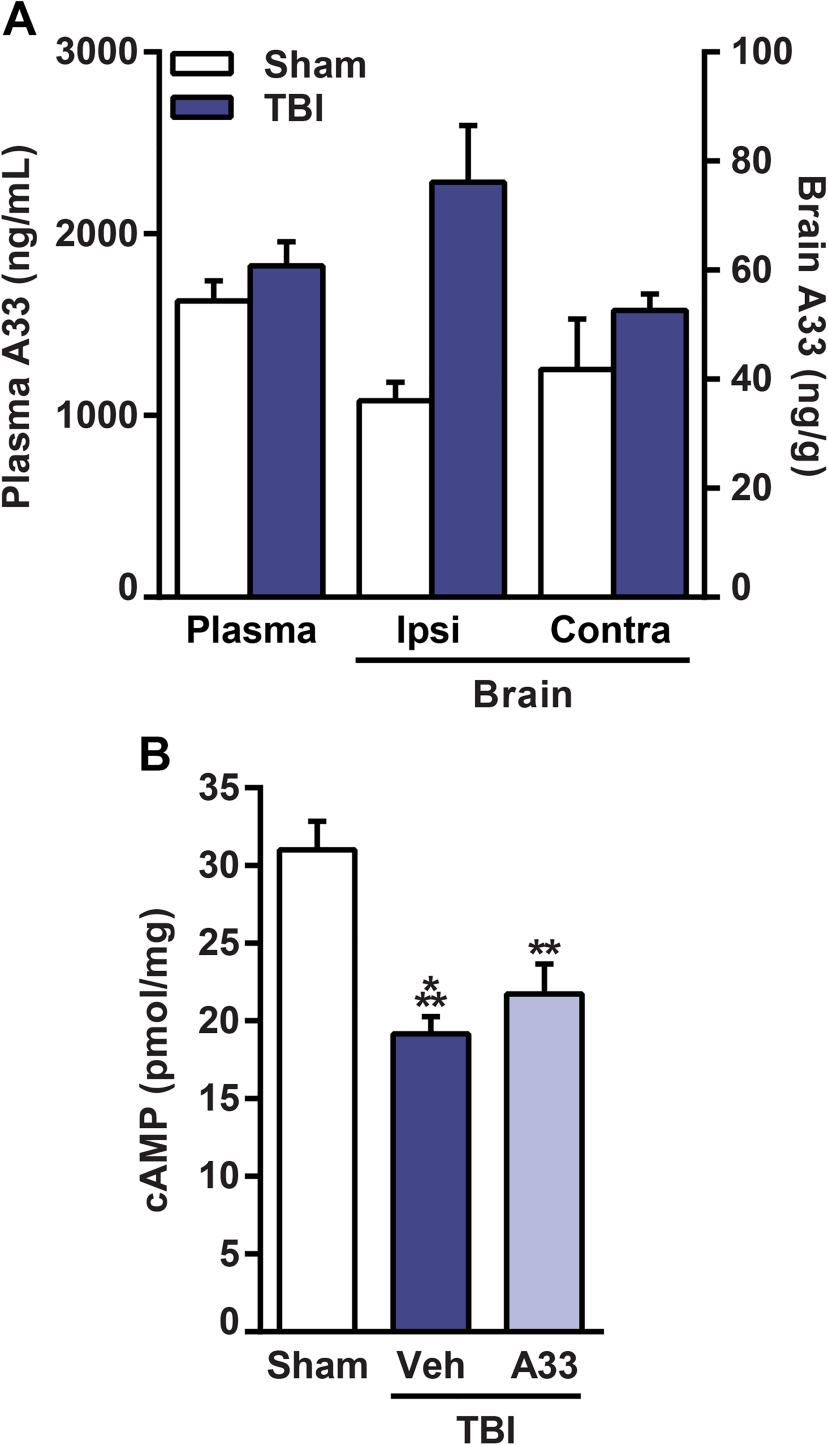
Distribution of A33 to the brain and plasma and effects on cAMP levels. **(A)** A33 levels were measured in the plasma and brain at 1 hr after A33 administration (0.3 mg/kg, i.p.). A33 levels were significantly higher in the brains of TBI animals as compared to sham animals (main effect of surgery: *F*_(1,8)_ = 11.986, *p* = 0.009 for brain A33 levels). Mean ± SEM, *n* = 3/group, two-way ANOVA with post-hoc Student-Newman-Keuls (brain), Student’s unpaired t-test (plasma). **(B)** Total cAMP levels were significantly decreased in the ipsilateral parietal cortex of vehicle- and A33-treated TBI animals as compared to sham animals. A33 treatment did not increase total cAMP in the cortex at 6 hrs post-injury as compared to vehicle-treated TBI animals. Mean ± SEM, *n* = 9-12/group, **p<0.01, ***p<0.001 vs. Sham, one-way ANOVA with post-hoc Student-Newman-Keuls.

To determine if A33 can reach concentrations in the rat brain capable of inhibiting PDE4B *in vivo*, we measured levels of A33 in the brain after sham and TBI surgery. Animals received sham or TBI surgery and were then treated with A33 (0.3 mg/kg, i.p.) at 30 min and 5 hrs post-surgery. This treatment schedule was chosen because we previously found that A33 treatment at 0.3 mg/kg (i.p.) significantly reduced TNF levels when administered at these acute time points after injury [50]. Additionally, the dose of A33 used in this study (0.3 mg/kg, i.p.) did not alter baseline cognitive measures when evaluated in sham rats and naïve mice, eliminating a potential confound when comparing to an effect on TBI animals [47, 50]. At 6 hrs post-surgery, brain and plasma samples were collected to measure A33 levels using tandem liquid chromatography—mass spectrometry. Plasma levels of A33 were similar between sham and TBI animals (**Fig 2A**). Interestingly, brain A33 levels were significantly higher in TBI animals as compared to sham animals (**Fig 2A**). Brain levels reached values that were 4 to 7-fold greater than the IC_50_ against PDE4B3 measured *in vitro* (27 nM or 10.4 ng/ml) [49, 50]. However, A33 had a relatively low brain distribution in the ipsilateral brain, with a brain/plasma ratio of 2.2 ± 0.2% and 4.2 ± 0.6% for sham and TBI animals, respectively. These results indicate that A33 can reach levels in the brain capable of inhibiting PDE4B.

To determine whether this treatment paradigm rescued basal cAMP deficits after TBI, the ipsilateral parietal cortex was evaluated at 6 hrs after sham or TBI surgery. This time point corresponds to previous studies demonstrating that cAMP is depressed in the injured cortex within 15 minutes after injury, and this depression persists for up to 3 days after moderate TBI [12, 56]. At 6 hrs after surgery, basal cAMP levels were significantly reduced in TBI animals as compared to sham animals. However, A33 treatment did not significantly increase cAMP levels in the injured cortex at 6 hrs post-injury (**Fig 2B**).

### Neutrophil accumulation is reduced with A33 treatment after TBI

**Fig 3:**
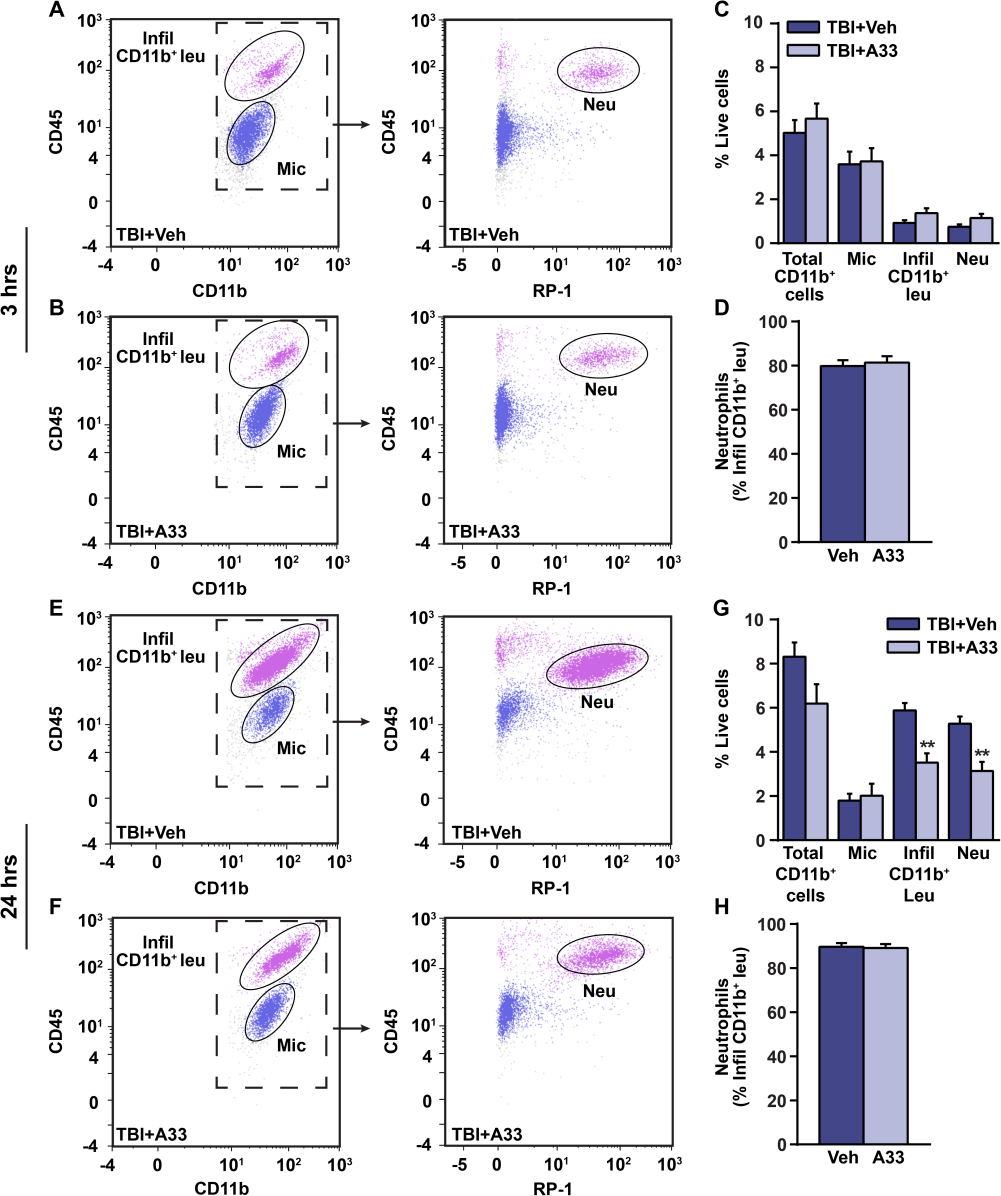
Neutrophil accumulation is reduced with A33 treatment at 24 hrs after TBI. Representative CD45/CD11b and CD45/RP-1 scatter plots from the ipsilateral parietal cortex of **(A)** vehicle-treated and **(B)** A33-treated animals at 3 hrs post-injury. CD45/RP-1 scatter plots were gated on total CD11b^+^ cells. **(C)** Quantification of total CD11b^+^ cells, microglia, infiltrating CD11b^+^ cells and neutrophils in vehicle-treated and A33-treated TBI animals at 3 hrs post-injury. **(D)** Percentage of infiltrating CD11b^+^ cells that are neutrophils (CD45^high^, CD11b^+^, RP-1^+^). Representative CD45/CD11b and CD45/RP-1 scatter plots at 24 hrs post-injury in **(E)** vehicle-treated and **(F)** A33-treated animals. **(G)** Quantification of total CD11b^+^ cells, microglia, infiltrating CD11b^+^ cells and neutrophils. **(H)** Percentage of infiltrating CD11b^+^ cells that are neutrophils at 24 hrs after TBI. Mean ± SEM, *n* = 10/group (3 hrs), *n* = 5/group (24 hrs), **p<0.01 vs. TBI+Vehicle, Student’s unpaired t-test. (RP-1 = rat neutrophil marker).

Neutrophils infiltrate the injured brain within hours after TBI, and accumulation of neutrophils is associated with increased neuronal damage and worsened outcome [28, 30, 31]. Given that PDE4B knockout mice have reduced neutrophil infiltration [26], we determined whether a PDE4B inhibitor could reduce neutrophil accumulation after TBI. To evaluate this, flow cytometry was used to determine neutrophil accumulation in the ipsilateral parietal cortex of vehicle and A33-treated TBI animals at 3 and 24 hrs after surgery (**Fig 3**). Neutrophils were identified as a subset of infiltrating myeloid-lineage cells (CD45^high^, CD11b^+^) using a rat neutrophil-specific marker, RP-1 [57, 58]. At 3 hrs post-injury, neutrophils were present in the injured cortex of both vehicle and A33-treated animals (**Fig 3A and 3B**), but were not reduced with A33 treatment at this time point (**Fig 3C**). At 3 hrs post-injury, neutrophils made up approximately 80% of infiltrating myeloid-lineage cells in both vehicle and A33-treated TBI animals (**Fig 3D**). When evaluated at 24 hrs after TBI, A33-treated TBI animals had significantly reduced infiltrating myeloid-lineage cells and neutrophils (**Fig 3E–3G**), with neutrophils making up approximately 90% of the infiltrating myeloid-lineage population in the injured brain (**Fig 3H**).

**Fig 4:**
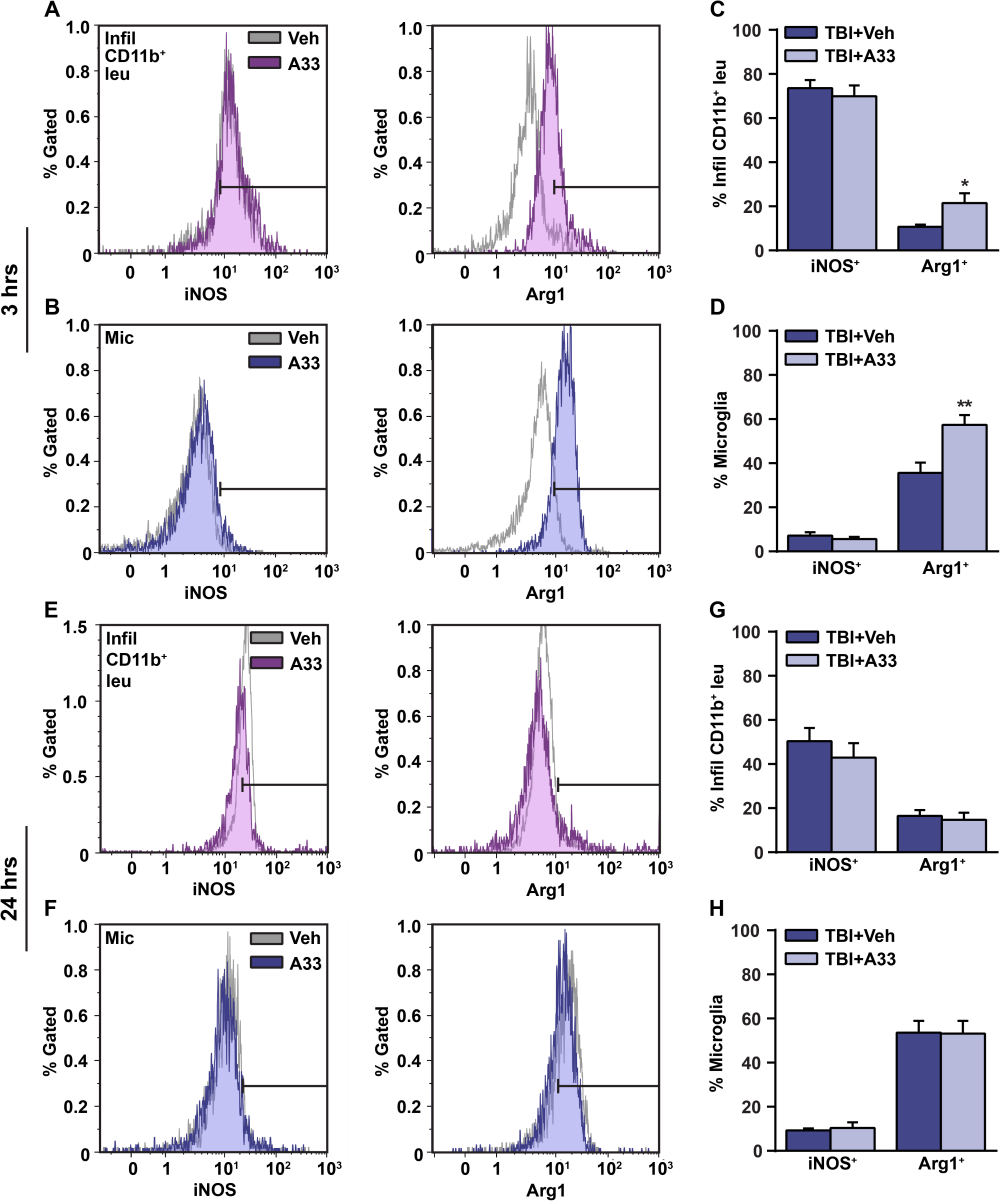
A33 treatment increased Arg1-expressing microglia and myeloid-lineage cells at 3 hrs after TBI. Representative histogram overlay of iNOS^+^ and Arg1^+^ **(A)** infiltrating CD11b^+^ cells and **(B)** microglia at 3 hrs post-injury. Quantification of iNOS and Arg1-expressing **(C)** infiltrating CD11b^+^ cells and **(D)** microglia. Representative histogram overlay of iNOS^+^ and Arg1^+^ **(E)** infiltrating CD11b^+^ cells and **(F)** microglia at 24 hrs post-injury. Quantification of iNOS and Arg1-expressing **(G)** infiltrating CD11b^+^ cells and **(H)** microglia at 24 hrs after TBI. Mean ± SEM, *n* = 10/group (3 hrs), *n* = 5/group (24 hrs), *p<0.05, **p<0.01 vs. TBI+Vehicle, Student’s unpaired t-test. (iNOS = inducible nitric oxide synthase, Arg1 = arginase 1).

In addition to measuring neutrophil accumulation, iNOS and Arg1 were used as markers to determine whether A33 treatment alters the activation state of microglia (CD45^low^, CD11b^+^) and infiltrating myeloid-lineage cells (CD45^high^, CD11b^+^) at 3 hrs and 24 hrs after TBI (**Fig 4**). At 3 hrs post-injury, acute PDE4B inhibition increased the percentage of Arg1-expressing infiltrating myeloid-lineage cells and microglia, however A33 treatment had no effect on iNOS levels in either cell population (**Fig 4A–4D**). At 24 hrs post-injury, A33 treatment had no effect on iNOS or Arg1-expressing microglia or infiltrating myeloid-lineage cells (**Fig 4E–4H**).

### Cortical contusion volume is reduced with A33 treatment

**Fig 5:**
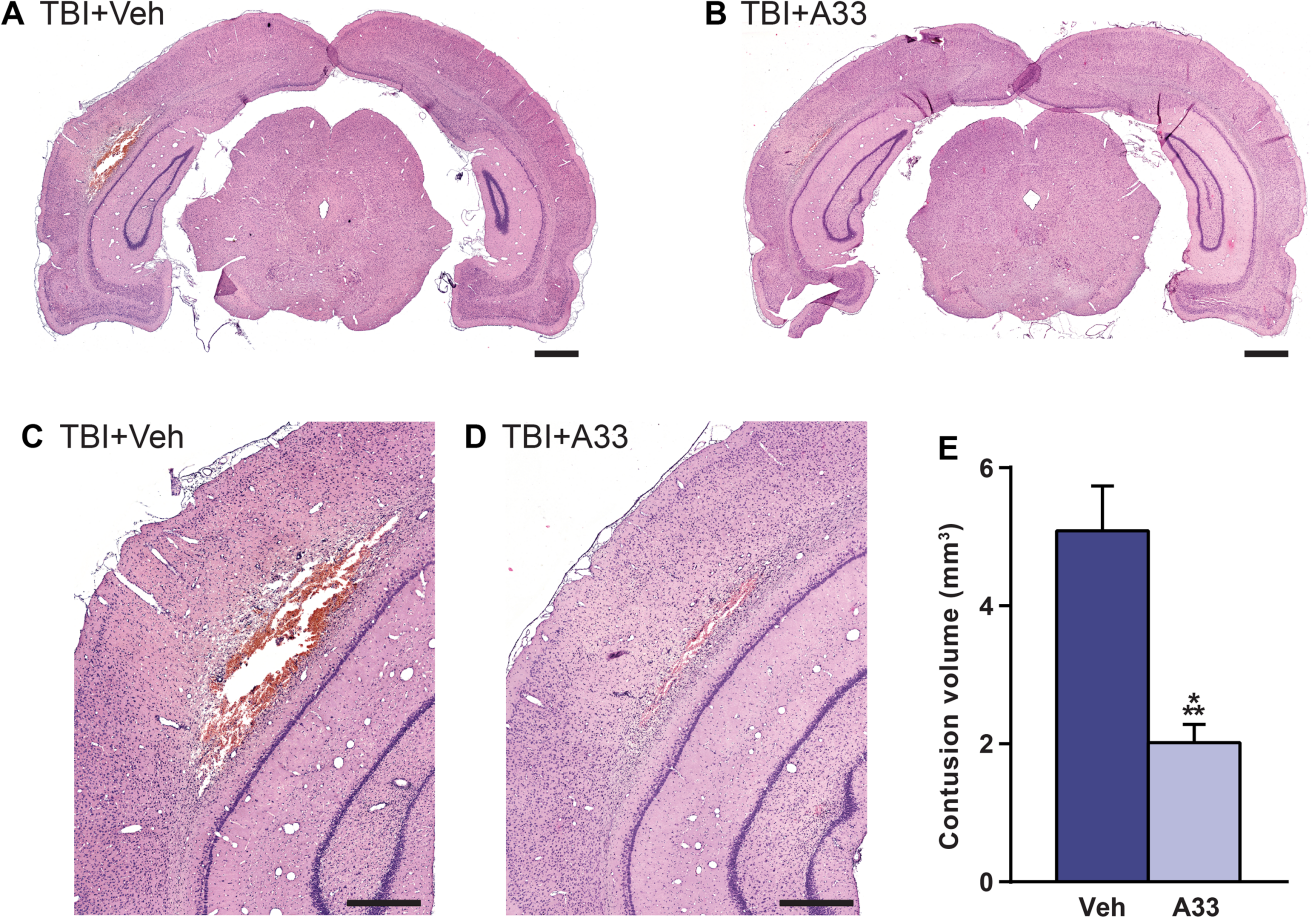
Acute PDE4B inhibition reduced cortical contusion volume at 3 days after TBI. Representative images of the ipsilateral parietal cortex stained with H&E from **(A,C)** vehicle-treated and **(B,D)** A33-treated animals at 3 days post-injury. Representative bregma level -6.3 mm. Scale bars **(A,B)** 1 mm and **(C,D)** 500 μm. **(E)** Quantification of contusion volume. Mean ± SEM, *n* = 10/group, ***p<0.001 vs. TBI+Vehicle, Student’s unpaired t-test.

While there are many factors that contribute to the developing contusion after brain injury, inflammation is one of the key driving forces [32, 59, 60]. Given that acute A33 treatment reduced inflammation, we next wanted to determine whether this treatment also reduced contusion volume. At 3 days after TBI, which corresponds to a time point when cortical contusion volume can be reliably quantified, animals were perfused and the brains were sectioned and stained with H&E to visualize the contused tissue [12, 21]. At 3 days post-injury, acute A33 treatment significantly reduced cortical contusion volume as compared to vehicle-treated TBI animals (**Fig 5**). This indicates that PDE4B inhibition with A33 can reduce a major pathology indicator when administered early after brain injury.

### Effects of a PDE4B inhibitor on behavioral recovery after TBI

Given that acute A33 treatment reduced inflammation and cortical contusion volume, we determined whether this treatment paradigm also reduced sensorimotor and learning and memory deficits after TBI. From 1 to 6 weeks post-surgery, animals were serially evaluated for sensorimotor and learning and memory deficits using the cylinder test, contextual fear conditioning, water maze and a working memory task.

**Fig 6:**
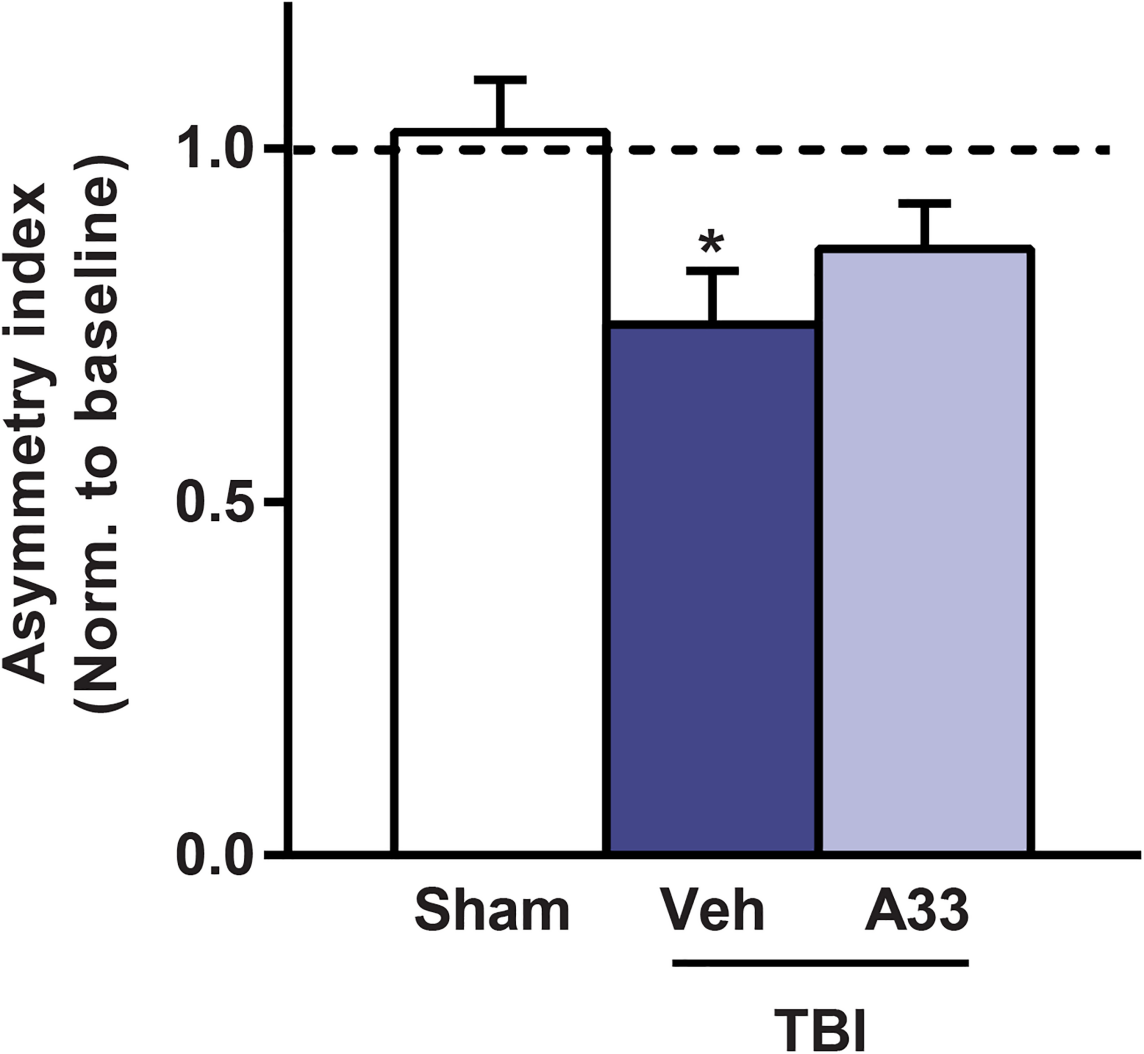
Acute PDE4B inhibition did not rescue sensorimotor deficits at 1 week after TBI. Animals were evaluated for spontaneous forelimb use asymmetry at 1 week after surgery using the cylinder task. Mean ± SEM, *n* = 12-14/group, *p<0.05 vs. Sham, one-way ANOVA with post-hoc Student-Newman-Keuls.

To evaluate sensorimotor deficits, we utilized the cylinder test. This task quantifies spontaneous asymmetrical forelimb use and reliably detects deficits in models that produce substantial unilateral damage, such as TBI and focal cerebral ischemia [16, 53, 61, 62]. At 1 week post-surgery, vehicle-treated TBI animals had significantly reduced contralateral forelimb use as compared to sham animals, as indicated by a decrease in asymmetry index (**Fig 6**). A33 treatment did not significantly improve contralateral forelimb use as compared to vehicle-treated TBI animals.

**Fig 7:**
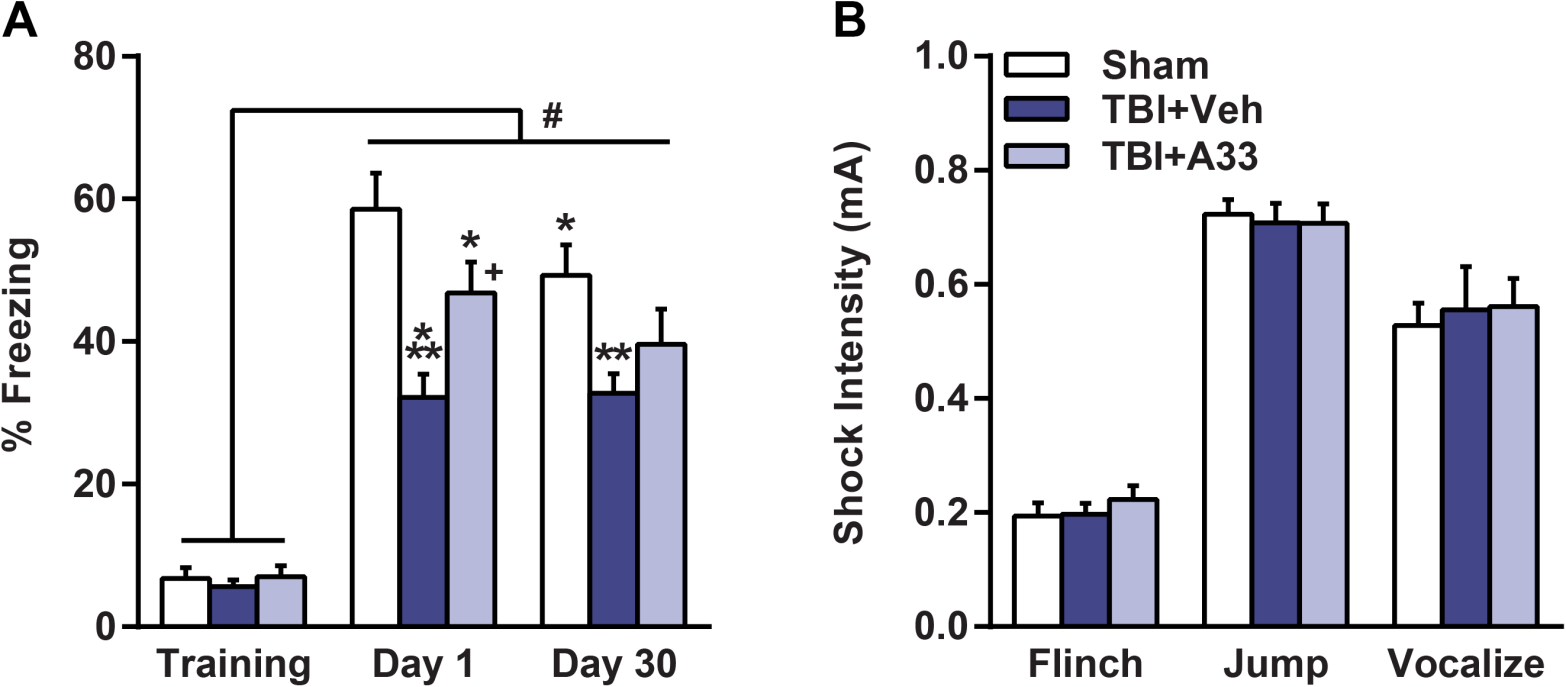
Acute PDE4B inhibition reduced contextual fear conditioning deficits at 2 weeks after TBI. **(A)** At 24 hrs and 1 month after training, animals were evaluated for contextual fear conditioning. At both 24 hrs and 1 month after training, vehicle-treated TBI animals froze significantly less than sham animals. A33-treated TBI animals froze significantly more than vehicle-treated TBI at 24 hrs, but not at 1 month, after training. A main effect of treatment (*F*_(2, 72)_ = 6.996, *p* = 0.003), a main effect of trial (*F*_(2, 72)_ = 169.111, *p*<0.001) and a significant interaction of treatment x trial (*F*_(4, 72)_ = 4.931, *p* = 0.001) were observed. *p<0.05, ***p<0.001 vs. Sham Day 1, **p<0.01 vs. Sham Day 30, ^*#*^*p*<0.001 vs. Training, ^*+*^*p*<0.01 vs. TBI+Vehicle Day 1, repeated-measures two-way ANOVA with post-hoc Student-Newman-Keuls. **(B)** Shock threshold was similar between all treatment groups. Mean ± SEM, *n* = 12-14/group, one-way ANOVA with post-hoc Student-Newman-Keuls.

Next, to determine whether this treatment paradigm reduced TBI-induced associative memory deficits, animals received contextual fear conditioning (**Fig 7**). There was no significant difference in baseline freezing between treatment groups. At 24 hrs after training, all animal groups exhibited significantly increased freezing. However, vehicle-treated TBI animals had significantly reduced contextual freezing as compared to sham animals and this was improved with A33 treatment at 24 hrs post-training (**Fig 7A**). When re-evaluated at 1 month after training, the improvement in contextual fear conditioning with A33 treatment was not maintained. Shock threshold assessment revealed no significant difference between treatment groups in the minimal shock intensity needed to elicit a flinch, jump or vocalization (**Fig 7B**).

**Fig 8:**
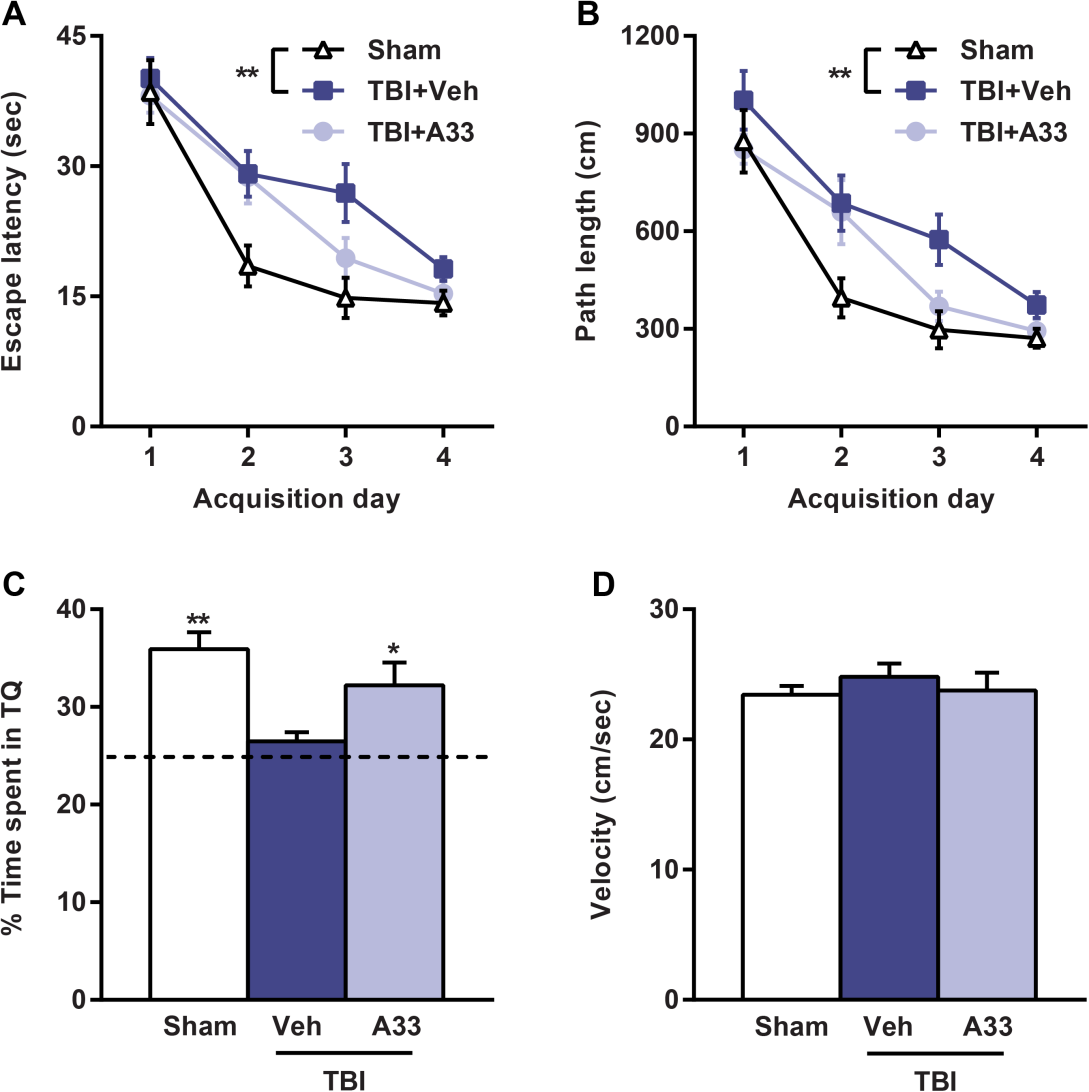
Acute PDE4B inhibition attenuated spatial memory deficits in the water maze task at 3 weeks post-TBI. During training, **(A)** escape latency and **(B)** path length were significantly increased in the vehicle-treated TBI animals as compared to sham (main effect of treatment: *F*_(2,111)_ = 5.446, *p* = 0.008 and *F*_(2,111)_ = 5.292, *p* = 0.010 for escape latency and path length, respectively), repeated-measures two-way ANOVA with post-hoc Student-Newman-Keuls. **(C)** During the probe trial, the percentage of time spent in the target quadrant (TQ) was significantly decreased in the vehicle-treated TBI animals as compared to sham and A33-treated TBI animals. **(D)** There was no difference in swim velocity between any of the treatment groups. Mean ± SEM, *n* = 12-14/group, *p<0.05, **p<0.01 vs. TBI+Vehicle, one-way ANOVA with post-hoc Student-Newman-Keuls.

To determine if acute PDE4B inhibition reduced TBI-induced spatial memory deficits, animals were evaluated using the water maze task. At 3 weeks post-surgery, vehicle-treated TBI animals had significantly impaired spatial learning acquisition as compared to sham animals, and this was not improved with A33 treatment (**Fig 8A and 8B**). However, memory retention was significantly improved with A33 treatment. During the probe trial, A33-treated TBI animals spent significantly more time in the target quadrant as compared to vehicle-treated TBI animals (**Fig 8C**). There was no significant difference in swim velocity between treatment groups (**Fig 8D**).

**Fig 9:**
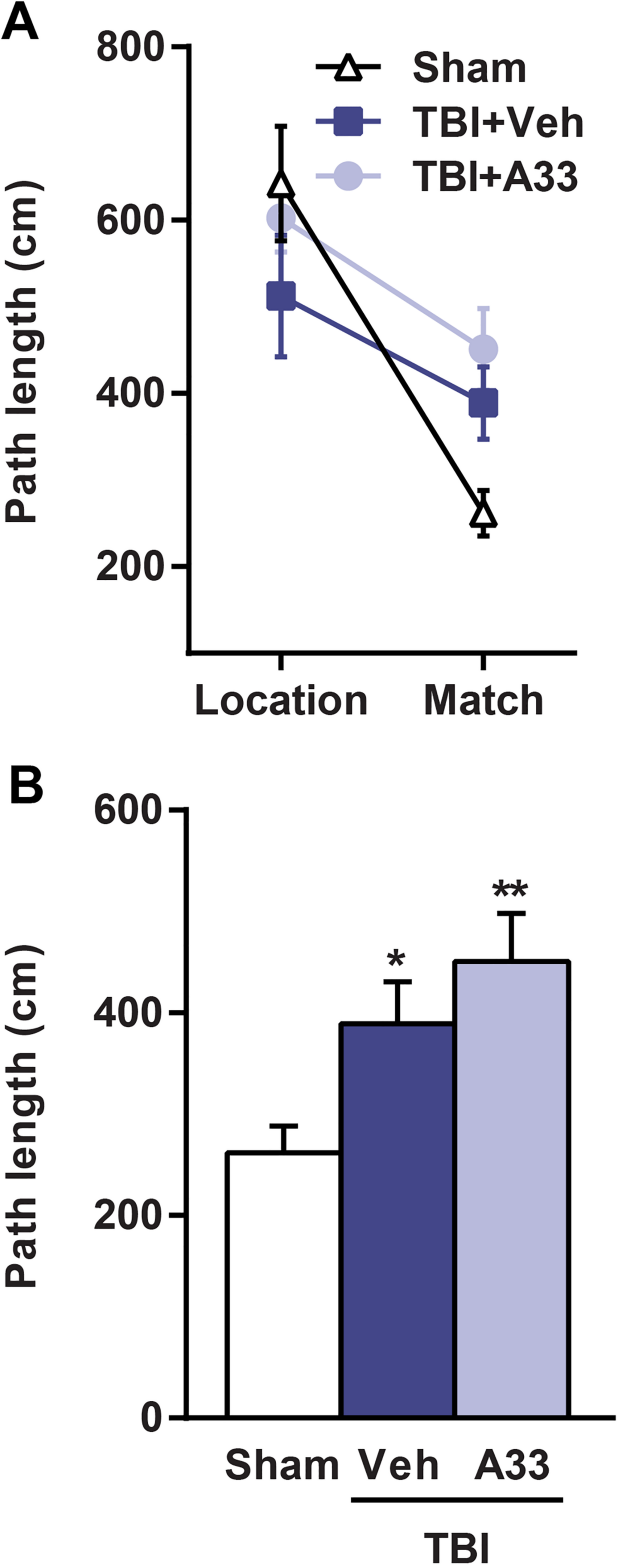
Acute PDE4B inhibition did not rescue spatial working memory deficits at 1 month post-TBI. **(A)** Path length from location and match trials. **(B)** Path length analysis of match trial. Both vehicle and A33-treated TBI animals had significantly impaired working memory, as measured by an increase in path length in the match trial as compared to sham animals. Mean ± SEM, *n* = 12-14/group, *p<0.05, **p<0.01 vs. Sham, one-way ANOVA with post-hoc Student-Newman-Keuls.

At 1 month post-surgery, the animals were evaluated for working memory [50, 63, 64]. Both vehicle and A33-treated TBI animals had significantly impaired working memory as compared to sham animals, however, A33 treatment did not rescue these working memory deficits (**Fig 9**). Together, these results indicate that acute PDE4B inhibition partially rescues long-term memory deficits, but does not improve short-term working memory or sensorimotor deficits after TBI.

### A33 treatment reduced TBI-induced atrophy and neuronal loss

**Fig 10:**
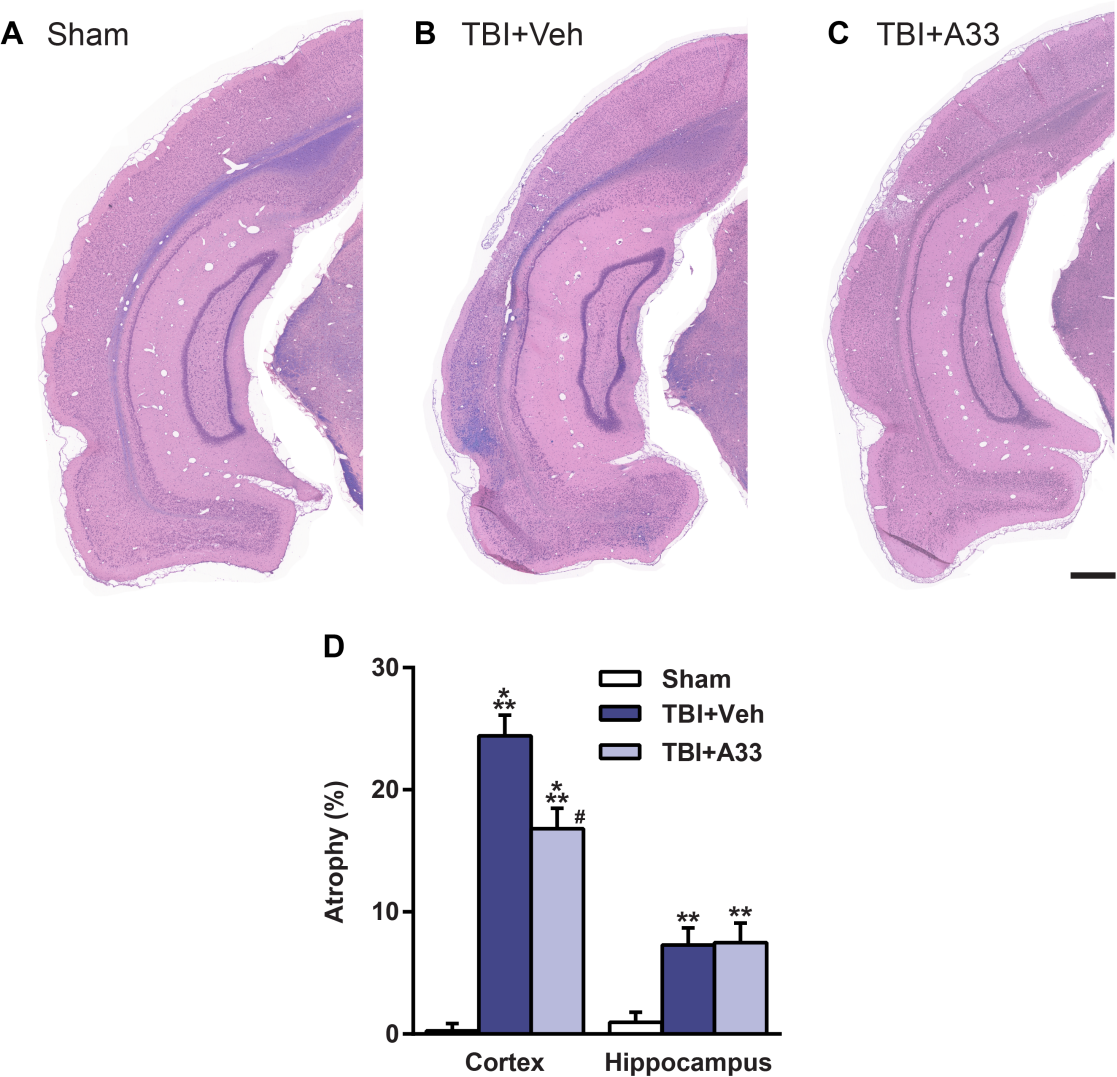
Acute PDE4B inhibition reduced cortical atrophy at 2 months post-injury. Representative images of the ipsilateral parietal cortex and hippocampus of **(A)** sham, **(B)** vehicle-treated and **(C)** A33-treated TBI animals stained with H&E plus Luxol fast blue. Representative images at -6.3 mm bregma, scale bar 500 μm. **(D)** Quantification of % atrophy. Both vehicle and A33-treated TBI animals had significantly increased cortical and hippocampal atrophy as compared to sham animals. A33-treated TBI animals had reduced cortical, but not hippocampal, atrophy as compared to vehicle-treated TBI animals. Mean ± SEM, *n* = 11-14/group, **p<0.01, ***p<0.001 vs. Sham, ^*#*^*p*<0.001 vs. TBI+Vehicle, one-way ANOVA with post-hoc Student-Newman-Keuls.

After TBI, the injured cortex and hippocampus are prone to progressive atrophy [65, 66]. To determine whether acute A33 treatment reduced cortical and hippocampal atrophy after TBI, animals were evaluated at 2 months post-surgery. At this time, both vehicle and A33-treated TBI animals had significant cortical and hippocampal atrophy as compared to sham animals (**Fig 10**). Cortical, but not hippocampal, atrophy was reduced in A33-treated TBI animals as compared to vehicle-treated TBI animals (**Fig 10**).

**Fig 11:**
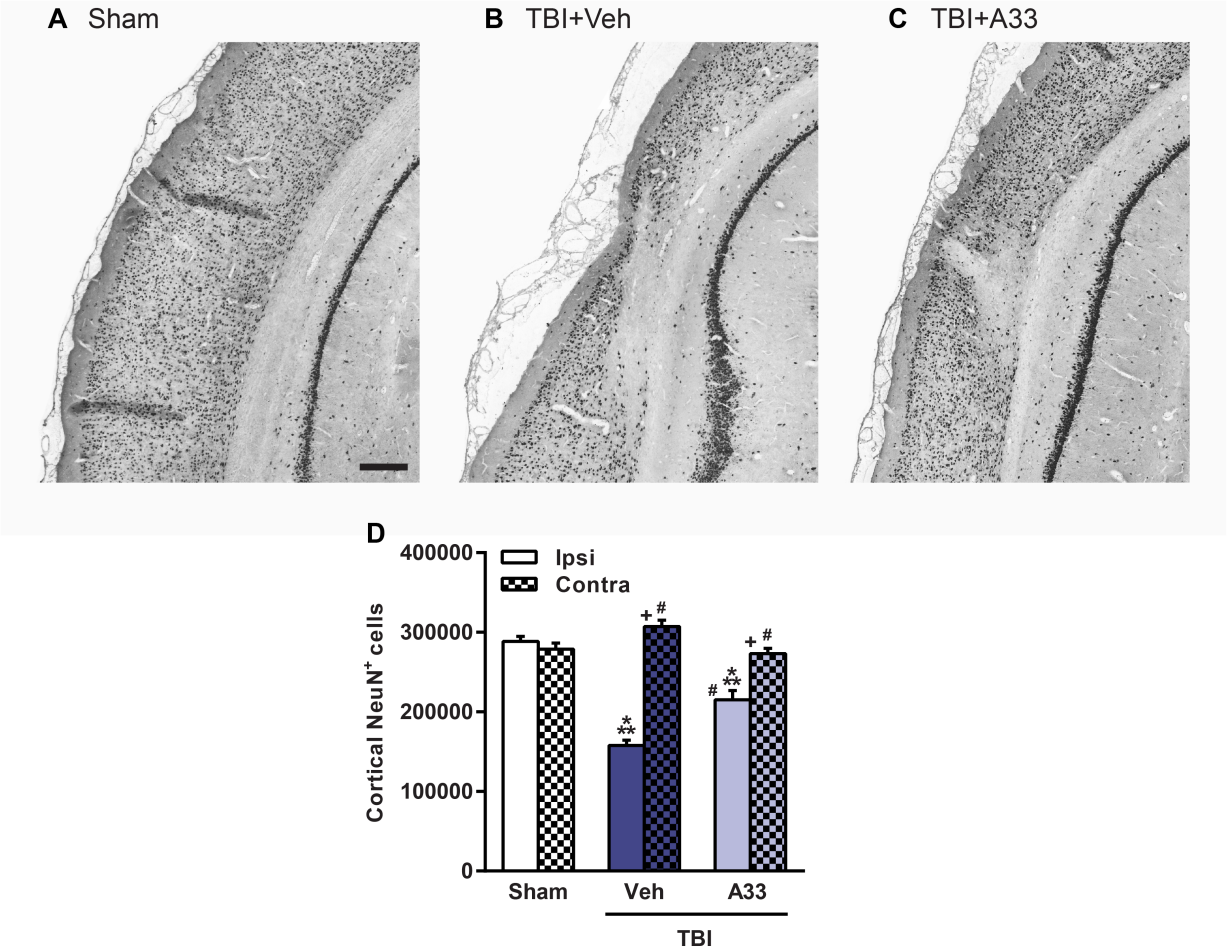
A33 treatment reduced neuronal loss in the pericontusional cortex at 2 months post-injury. Representative images of the ipsilateral parietal cortex of **(A)** sham, **(B)** vehicle-treated and **(C)** A33-treated TBI animals, immunolabeled for mature neurons using NeuN. Representative images at -5.3 mm bregma, scale bar 250 μm. **(D)** Quantification of NeuN^+^ cells in the ipsilateral and contralateral parietal cortex. The number of NeuN^+^ cells were significantly reduced in the ipsilateral parietal cortex in both vehicle and A33-treated TBI animals as compared to sham animals. A33 treatment rescued neuronal loss in the pericontusional cortex as compared to vehicle-treated TBI animals. (main effect of treatment: *F*_(2, 64)_ = 19.21, *p*<0.0001; main effect of region (ipsi vs. contra): *F*_(1, 64)_ = 93.68, *p*<0.0001; interaction of treatment x region: *F*_(2, 64)_ = 43.89, *p*<0.0001.) Mean ± SEM, *n* = 10-13/group, ***p<0.001 vs. Ipsi/Contra Sham, ^*#*^*p*<0.001 vs. Ipsi TBI+Vehicle, ^*+*^*p*<0.001 vs. Ipsi TBI+A33, two-way ANOVA with post-hoc Student-Newman-Keuls.

**Fig 12:**
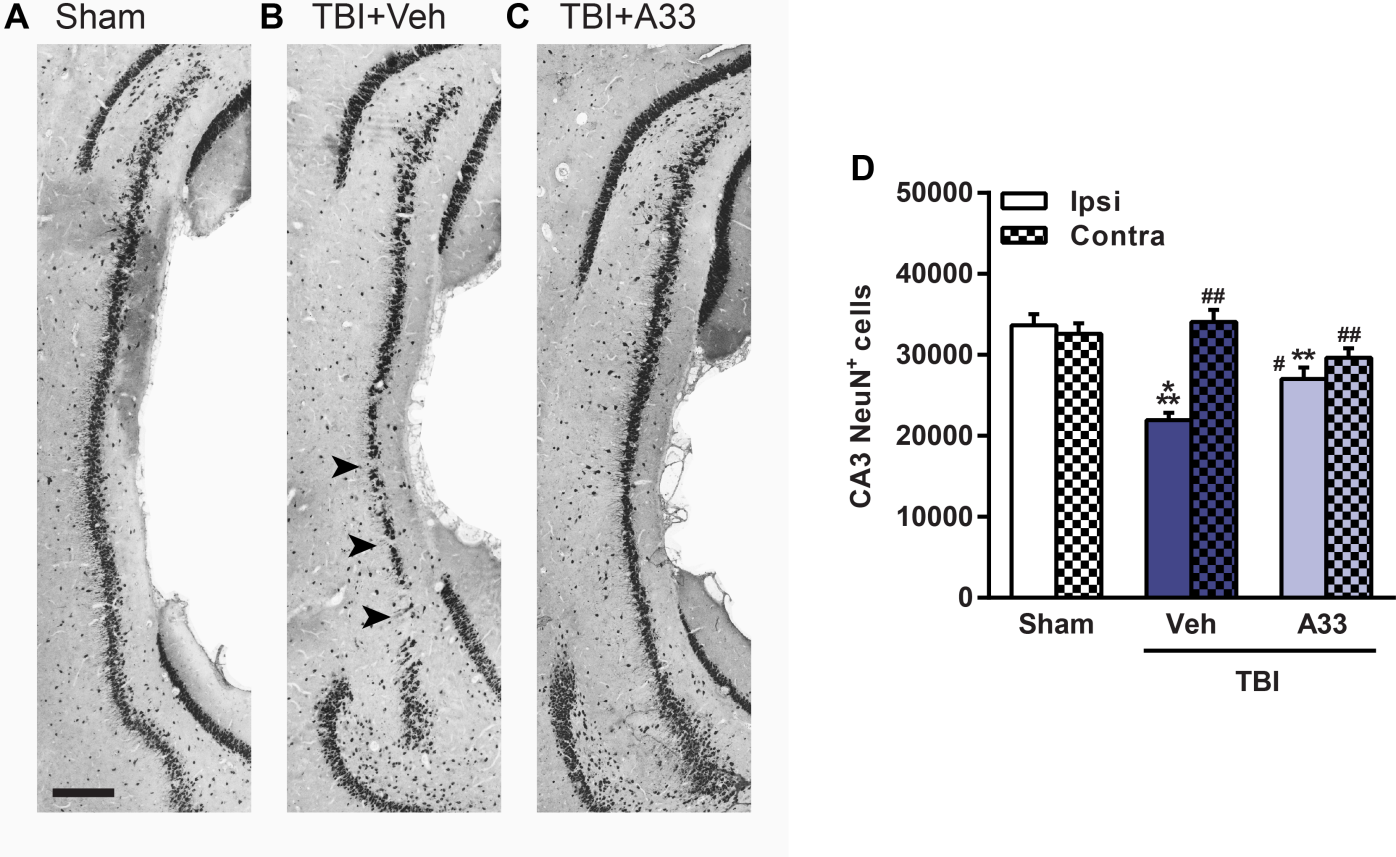
Treatment with a PDE4B inhibitor reduced neuronal loss in the hippocampal CA3 region at 2 months post-injury. Representative images of the ipsilateral CA3 region of the hippocampus immunolabeled for mature neurons using NeuN in **(A)** sham, **(B)** vehicle-treated and **(C)** A33-treated TBI animals. Representative images at -5.3 mm bregma, scale bar 250 μm. **(D)** Quantification of NeuN^+^ cells in the ipsilateral and contralateral CA3 region of the hippocampus. Vehicle and A33-treated TBI animals had significantly reduced NeuN^+^ cells in the ipsilateral hippocampal CA3 region as compared to sham animals. A33 treatment partially rescued neuronal loss in the ipsilateral hippocampal CA3 region as compared to vehicle-treated TBI animals. (main effect of treatment: *F*_(2, 64)_ = 8.977, *p* = 0.0004; main effect of region (ipsi vs. contra): *F*_(1, 64)_ = 18.45, *p*<0.0001; interaction of treatment x region: *F*_(2, 64)_ = 13.38, *p*<0.0001.) Mean ± SEM, *n* = 10-13/group, **p<0.01, ***p<0.001 vs. Ipsi/Contra Sham, ^*#*^*p*<0.01, ^*##*^*p*<0.001 vs. Ipsi TBI+Vehicle, two-way ANOVA with post-hoc Student-Newman-Keuls.

After TBI, the parietal cortex and CA3 region of the hippocampus are particularly vulnerable to neuronal loss [12, 67]. When quantifying neuronal loss in the pericontusional cortex (**Fig 11**) and CA3 region of the hippocampus (**Fig 12**), both vehicle and A33-treated TBI animals had a significant reduction in NeuN^+^ cells as compared to sham animals. A33 treatment partially reduced neuronal loss in both the pericontusional cortex (**Fig 11**) and CA3 region of the hippocampus (**Fig 12**). These results suggest that a PDE4B inhibitor delivered acutely after TBI reduces neuronal loss and atrophy at chronic time points after injury.

## Discussion

Approximately 80% of TBI survivors will develop learning and memory deficits [4]. Therapeutics targeting the TBI-induced inflammatory response have yielded promising results in preclinical studies for reducing neuronal loss and memory deficits [29, 68–70]. In this study, we set out to determine whether a PDE4B-selective inhibitor, A33, would reduce inflammation and improve outcome after TBI. The results of this study demonstrate that acute treatment with a PDE4B-selective inhibitor, A33, reduces inflammation, pathology and memory deficits after TBI. The partial rescue in memory deficits, atrophy and neuronal loss after TBI is suggestive that the therapeutic benefits of acute PDE4B inhibition persist for weeks after treatment.

In 2009, Naganuma and colleagues reported the discovery of a PDE4B-selective inhibitor, compound 33 [48]. Prior to the discovery of compound 33, now termed A33, the high structural similarity between PDE4B and PDE4D precluded the development of PDE4B-selective inhibitors [48, 49]. In this seminal paper, Naganuma *et al*. demonstrated that A33 was not only highly selective for PDE4B, but also had good pharmacokinetic properties, with 85% bioavailability in mice when administered orally [48]. However, whether systemic administration of A33 reached levels capable of inhibiting PDE4B in the rat brain acutely after TBI was unknown. Thus, we measured A33 in the brain at 6 hrs after sham or TBI surgery. At 6 hrs post-surgery, A33 reached concentrations 4 to 7-fold above the IC_50_ for PDE4B. Furthermore, A33 levels were nearly 2-fold higher in the injured brain than in the uninjured. The increased distribution of A33 in the acutely injured brain are contrary to what was previously observed in chronic A33-treated animals, where there was no significant difference in brain A33 levels in sham versus TBI animals [50]. The increased levels of A33 in the injured brain are likely due to blood-brain barrier (BBB) breakdown at this early time point after TBI [71]. However, the brain/plasma ratio was relatively low in both sham and TBI animals, possibly due to a high efflux of A33 (efflux ratio = 14.4) [50]. One caveat of these results is that the animals were not perfused prior to tissue collection. Therefore, when measuring brain levels of A33, both residual blood and brain tissue were evaluated. Studies are currently in progress to improve the pharmacokinetics of A33, specifically regarding chemical modifications that will increase A33 distribution in the brain.

In addition to examining the distribution of A33 in the injured brain, we tested whether this PDE4B-selective inhibitor was capable of augmenting cAMP levels after TBI. A recent study by Zhang *et al*. demonstrated that A33 augments cAMP levels in isoproterenol-stimulated hippocampal cells [47]. In the context of TBI, we previously reported that the pan-PDE4 inhibitor, rolipram, rescued basal cAMP deficits [12]. However, contrary to those previous studies, we found that A33 did not significantly increase basal cAMP levels after TBI. This negative result is likely because rolipram inhibits all PDE4 subfamilies, whereas A33 is selective for PDE4B. While this study focused on the PDE4B subfamily, we previously reported that PDE4D2 expression and PDE4A5 phosphorylation are significantly increased in the injured cortex acutely after TBI [45]. Thus, PDE4A and 4D subfamilies may have contributed to the decrease in basal cAMP levels after TBI. Furthermore, previous studies have demonstrated that PDE4B regulates cAMP levels near the plasma membrane and in mitochondria, but not in the cytosol [72, 73]. In contrast, PDE4D predominantly hydrolyzes cytosolic cAMP [72]. Consequently, the cAMP assay used in this study may not have been sensitive enough to detect PDE4B-mediated changes in basal cAMP levels after TBI. Future studies evaluating PDE4B inhibitors would benefit from the use of cAMP-sensitive fluorescent sensors to detect localized changes of cAMP [72].

The short PDE4B isoform, PDE4B2, has been implicated in regulating the activation of inflammatory cells, such as macrophages, microglia and neutrophils [15, 42, 43]. In a previous study, we found that PDE4B2 is significantly increased in the injured cortex and hippocampus as early as 30 min post-injury, and remained elevated up to 24 hrs after TBI [45, 74]. However, whether PDE4B2 was upregulated in particular inflammatory cell populations in the injured cortex had not been determined. Using flow cytometry, we found that PDE4B2-expressing CD11b^+^ cells were significantly increased in the injured cortex at 24 hrs after TBI. This increase in PDE4B2^+^ cells was due to an increase in both PDE4B2-expressing microglia and infiltrating myeloid-lineage cells. However, the mechanism that regulates this increase in PDE4B2 expression in inflammatory cells is unknown. One possible mechanism for the TBI-induced increase in PDE4B2 expression in microglia and infiltrating myeloid-lineage cells is through toll-like receptor 4 (TLR4) signaling. Several studies have reported that TLR4 signaling, via LPS stimulation, increases expression of PDE4B2 [25, 43, 75]. After TBI, the time course of TLR4 upregulation parallels the acute increase observed in PDE4B2 expression, and TLR4 has been shown to localize to microglia and neutrophils acutely after injury [76–78]. Furthermore, TLR4 knockout mice have reduced inflammation and improved outcome after TBI [79]. Altogether, these findings suggest that additional studies delineating the mechanisms that regulate PDE4B2 expression could provide additional targets, such as TLR4, for reducing inflammation and improving recovery after TBI.

After TBI, neutrophil accumulation has been associated with histopathological damage and poor recovery [28, 30, 31]. Treatment strategies aimed at reducing neutrophil accumulation after TBI have yielded positive results, with improved neuronal survival and behavioral recovery [32, 33]. Given that PDE4B knockout and A33-treated mice have reduced neutrophil accumulation in systemic models of inflammation, we tested whether A33 treatment reduced neutrophil accumulation after TBI [26, 48]. Using flow cytometry, we determined whether A33 treatment reduced neutrophil accumulation at 3 and 24 hrs after TBI, which corresponds to the early and peak infiltration time points after injury [27]. At 24 hrs, but not 3 hrs after injury, A33 treatment significantly reduced neutrophil accumulation. The reduction in peak neutrophil accumulation at 24 hrs but not at 3 hrs suggests that the immediate influx of neutrophils after TBI may not be dependent on PDE4B-regulated pathways. While the exact mechanism responsible for the A33-mediated reduction in neutrophil accumulation at 24 hrs after TBI is unknown, studies using PDE4B knockout mice suggest that these findings are due to decreased CD18 expression in circulating neutrophils [26]. CD18 is an important mediator of neutrophil-endothelial cell adhesion and infiltration, and expression of CD18, like PDE4B2, is regulated by TLR4 signaling [80–82]. Overall, these results suggest that PDE4B is a possible therapeutic target for reducing peak neutrophil accumulation after TBI.

Pan-PDE4 inhibitors have been found to regulate the activation state of microglia and macrophages during the early inflammatory response after CNS injury [34, 38]. Two commonly used markers of classical (M1) and alternative (M2) activation are iNOS and Arg1, respectively [38, 39, 41, 83, 84]. Therefore, in this study we used iNOS and Arg1 as markers to determine whether the PDE4B-selective inhibitor, A33, alters the activation state of microglia and myeloid-lineage cells after TBI. At 3 hrs, but not 24 hrs after injury, A33 treatment significantly increased the percentage of Arg1-expressing microglia and infiltrating myeloid-lineage cells. The upregulation of Arg1 at 3 hrs, but not 24 hrs, in the A33-treated TBI animals suggests that this effect does not persist at longer time points after A33 treatment, given that the final dose was administered at 5 hrs after TBI. Due to the emerging evidence on the complexity of M1/M2 phenotypes, we cannot definitively conclude whether acute PDE4B inhibition induces an M2 activation state after TBI [85–88]. Nevertheless, the reduction in neutrophil accumulation at 24 hrs post-injury, which was preceded by an early increase in Arg1-expressing microglia and infiltrating myeloid-lineage cells, is suggestive of a more anti-inflammatory/pro-reparative environment with A33 treatment.

While we previously demonstrated that acute PDE4B inhibition reduces TNF levels after TBI, future studies will benefit from a more comprehensive assessment of changes in the cytokine and chemokine profile with PDE4B inhibition [50]. Several studies have demonstrated that PDE4 negatively regulates cAMP levels in pathways that control the expression and anti-inflammatory effects of interleukin 4 (IL-4), IL-13 and IL-10 [37, 52, 89, 90]. These cytokines, as well as Arg1, are considered M2 markers in microglia and macrophages [41, 91, 92]. Moreover, these markers are regulated by cAMP-Stat6 (signal transducer and activator of transcription 6) signaling pathways, suggesting that PDE4B inhibition may induce expression of multiple M2 markers [36, 93]. Overall, a greater understanding of the anti-inflammatory actions of PDE4B inhibitors would have therapeutic implications not just for TBI, but also for CNS injuries and diseases where PDE4B is upregulated.

Cerebral contusions and hematomas are a common type of mass lesion after TBI [59, 94]. These lesions can be life-threatening and lead to life-long disabilities if not properly mitigated, which in clinical settings is often accomplished through surgical intervention [95, 96]. Preclinical studies have implicated the acute inflammatory response in exacerbating cerebral contusions and hematomas [60, 97]. This inflammatory response further drives BBB breakdown, increases hemorrhage and exacerbates the developing contusions [32, 71]. Thus, a therapeutic that has the ability to attenuate the acute inflammatory response may also reduce the extent of hemorrhage and contusion after TBI. At 3 days post-injury, we found that A33 treatment significantly reduced contusion volume. These results differed from what we previously observed when evaluating the pan-PDE4 inhibitor, rolipram, acutely after TBI [21, 22]. While pan-PDE4 inhibition worsened cortical contusions acutely after TBI, acute PDE4B inhibition reduced cortical contusions [21, 22]. This reduction in contusion volume is suggestive of a reduction in BBB breakdown in A33-treated TBI animals. BBB breakdown is seen within minutes to hours after TBI and is associated with neutrophil infiltration [27, 71, 98, 99]. Thus, the reduction in both neutrophil accumulation and cortical contusion volume in A33-treated TBI animals suggests that PDE4B may be a biological target for reducing BBB breakdown after TBI. One possible mechanism for this reduction in BBB breakdown may be through upregulation of endothelial adherens junctions, such as VE-cad (vascular endothelial cadherin) [100]. VE-cad is essential for proper BBB function, and expression of VE-cad is positively regulated by cAMP signaling [101, 102]. Furthermore, augmented VE-cad levels are associated with reduced BBB breakdown acutely after TBI [103]. Regardless of the mechanism, the reduction in cortical contusion volume in this study supports the use of PDE4B inhibitors as a treatment for attenuating cerebral contusion progression after TBI.

Learning and memory deficits are common long-term consequences in both experimental and clinical TBI [2, 4, 53, 64]. Previously, we reported that delayed A33 treatment reduced memory deficits at 3 months post-TBI, but the treatment was limited to 30 min prior to training in the learning task [50]. In this study, we tested whether acute A33 treatment reduced TBI-induced memory deficits several weeks after the treatment period. We found that acute A33 treatment partially rescued memory recall in the water maze task. This improvement in recent memory recall was further supported by a partial rescue in contextual fear conditioning deficits at 24 hrs post-training. However, the improvement in contextual fear conditioning was not persistent, and remote recall at 1 month post-training (6 weeks after surgery) was not improved with A33-treatment. These results suggest that the memory trace during recall and consolidation at the 24 hr testing time point was not sufficient to sustain the fear memory at 1 month after training [104, 105]. This long-term cognitive decline may be due in part to the progressive hippocampal atrophy caused by TBI, which was not mitigated with acute A33 treatment [66, 106]. Acute A33 treatment did significantly reduce cortical atrophy, as well as neuronal loss in the hippocampal CA3 region and pericontusional cortex. Although the reduction in hippocampal CA3 neuronal loss may have partially reduced TBI-induced memory deficits, this neuronal sparing was not enough to demonstrably reduce hippocampal atrophy. The apparent discrepancy between the improved CA3 neuronal survival and unaffected hippocampal atrophy may be due to the combined reduction in the number and volume of remaining neurons in the hippocampus [66]. While we quantified the number of remaining neurons in the hippocampal CA3 region, we did not measure neurite length and synaptic density. Several studies have demonstrated that TBI causes a decrease in dendrite length and synaptic density in the hippocampus [107–109]. Therefore, the partial rescue in the number of hippocampal CA3 neurons may have been surpassed by the atrophy of the remaining neurons in the A33-treated TBI animals. Future studies attempting to reduce hippocampal atrophy may benefit from a more extended treatment course with A33 after injury. Altogether, this study suggests that acute A33 treatment is capable of improving recent memory recall at chronic time points after TBI, possibly through a partial reduction in CA3 neuronal loss.

The neuroprotective benefits of pan-PDE4 inhibitors are well documented in models of TBI, SCI and cerebral ischemia [12, 13, 110]. After TBI, neuronal damage in the injured cortex and hippocampus can be seen within minutes after injury and progresses to measurable neuronal loss within hours [27, 111, 112]. In this study, we examined some of the anti-inflammatory actions of A33 treatment as a potential mechanism for reducing neuronal loss and memory deficits after TBI. However, an unexplored mechanism in which A33 may prevent neuronal loss after TBI is through cell survival pathways. Previously, we reported that chronic A33 treatment significantly increased basal levels of phosphorylated cyclic AMP-response element-binding protein (CREB) in the injured hippocampus [50]. CREB acts downstream of cAMP and increased levels of phosopho-CREB (Ser133) are often used as a surrogate measure to evaluate increases in cAMP signaling [64, 113]. In cell survival pathways, phosopho-CREB can activate transcription of anti-apoptotic genes, such as *bcl-2* (B-cell lymphoma 2) [114]. Furthermore, preclinical TBI studies have demonstrated that overexpression of Bcl-2 attenuates both cortical and hippocampal CA3 neuronal loss [115, 116]. However, in this study we did not examine whether pro-survival pathways were augmented in neurons when treated with A33 acutely after TBI. One potential mechanism in which increasing cAMP, via PDE4B inhibition, may promote neuronal survival is through elevated mitochondrial CREB activation [117]. Mitochondria play a major role in regulating apoptotic signaling, and some of these anti-apoptotic pathways involve mitochondrial CREB-mediated activation of cell survival pathways [114]. In support of this hypothesis, studies have demonstrated that PDE4B2 and an associated anchoring protein, DISC1 (disrupted in schizophrenia 1), colocalize in neuronal mitochondria [73, 118]. Furthermore, we previously found that PDE4B2 is upregulated in cortical and hippocampal dendrites early after brain injury [45, 74]. However, whether the TBI-induced increase in neuronal PDE4B2 expression occurs within mitochondria is unknown. Moreover, whether PDE4B2 negatively regulates CREB-mediated neuronal survival pathways has not yet been established. With the development of PDE4B-selective inhibitors, such as A33, future studies could explore the role of PDE4B as a dual anti-inflammatory/pro-survival target for a wide array of neurological injuries and diseases.

This is the first study to investigate the therapeutic potential of acute treatment with a PDE4B-selective inhibitor after TBI. While we found that A33 treatment reduced several TBI-induced pathologies, there are several limitations of the current study. The early administration of A33 at 30 min post-injury is a significant limitation with regards to clinical translation. This treatment time was chosen to optimize observing a potential effect of A33, given the rapid onset of inflammation after TBI [27, 119–121]. However, this time point is not feasible for many TBI patients. On average, the earliest time point a TBI patient is evaluated and treated by a physician varies between 1–6 hrs after injury [122, 123]. Therefore, future studies are needed to evaluate the therapeutic time window for acute A33 treatment after TBI. Another limitation of the current study is the lack of efficacy of A33 to improve several cognitive measures evaluated in this study. While acute A33 treatment rescued deficits in contextual fear conditioning and water maze retention, neither working memory nor contextual fear conditioning at 1 month post-training were improved. The lack of efficacy could have been due to a suboptimal dosing schedule. Assessing a dose-response curve, as well as evaluating other dosing schedules, are necessary to increase the likelihood for successful clinical translation in future studies. Another possible explanation for the lack of efficacy observed in some of the behavioral measures is the possible contribution of other PDE4 subfamilies to TBI-induced cognitive deficits. We previously reported that phospho-PDE4A5 and PDE4D2 are elevated in the injured cortex and hippocampus acutely after TBI [45, 74]. Previous studies have demonstrated a potential role for these PDE4 isoforms in cognition. Li *et al*. reported that knockout of PDE4D enhanced water maze retention and novel object recognition [124]. Additionally, a study from Havekes *et al*. demonstrated that augmenting levels of PDE4A5 in mice impaired both contextual fear conditioning and novel object recognition [125]. Therefore, other PDE4 subfamilies may have contributed to the cognitive deficits caused by TBI. Future studies evaluating the therapeutic window and dose-response curve of acute A33 treatment, as well as investigating the contribution of other PDE4 isoforms to TBI pathology, will clarify the potential of PDE4B-selective inhibitors as acute therapeutics for TBI.

Over the last 40 years, there has been extensive development in the use of pan-PDE4 inhibitors as anti-inflammatory treatments [126, 127]. Currently, there are two FDA-approved pan-PDE4 inhibitors available for clinical use, Roflumilast and Apremilast [128, 129]. However, lack of specificity for individual PDE4 subfamilies has hindered their use after TBI. Overall, this study demonstrated that a PDE4B-selective inhibitor, A33, can reduce inflammation, histopathology and memory deficits after TBI. The multifactorial and beneficial effects observed with A33 treatment support the use of PDE4B inhibitors as an anti-inflammatory, and possibly neuroprotective, treatment strategy for TBI.

## References

[1] Faul M, Xu L, Wald MM, and Coronado VG. Traumatic brain injury in the united states: Emergency department visits, hospitalizations and deaths 2002–2006. Centers for Disease Control and Prevention, National Center for Injury Prevention and Control 2010.

[2] Zaloshnja E, Miller T, Langlois JA, and Selassie AW. Prevalence of long-term disability from traumatic brain injury in the civilian population of the united states, 2005. J Head Trauma Rehabil. 2008; 23(6):394–400. doi: 10.1097/01.HTR.0000341435.52004.ac 19033832 10.1097/01.HTR.0000341435.52004.ac

[3] Faul M and Coronado V. Epidemiology of traumatic brain injury. Handb Clin Neurol. 2015; 127:3–13. doi: 10.1016/B978-0-444-52892-6.00001-5 25702206 10.1016/B978-0-444-52892-6.00001-5

[4] Lew HL, Poole JH, Guillory SB, Salerno RM, Leskin G, and Sigford B. Persistent problems after traumatic brain injury: The need for long-term follow-up and coordinated care. J Rehabil Res Dev. 2006; 43(2):vii–x. 16847779 10.1682/jrrd.2006.05.0054

[5] Whitnall L, McMillan TM, Murray GD, and Teasdale GM. Disability in young people and adults after head injury: 5–7 year follow up of a prospective cohort study. J Neurol Neurosurg Psychiatry. 2006; 77(5):640–645. doi: 10.1136/jnnp.2005.078246 16614025 10.1136/jnnp.2005.078246 PMC2117429

[6] Moretti L, Cristofori I, Weaver SM, Chau A, Portelli JN, and Grafman J. Cognitive decline in older adults with a history of traumatic brain injury. Lancet Neurol. 2012; 11. 10.1016/S1474-4422(12)70226-0 23153408

[7] Cuthbert JP, Harrison-Felix C, Corrigan JD, Bell JM, Haarbauer-Krupa JK, and Miller AC. Unemployment in the united states after traumatic brain injury for working-age individuals: Prevalence and associated factors 2 years postinjury. J Head Trauma Rehabil. 2015; 30(3):160–74. doi: 10.1097/HTR.0000000000000090 25955703 10.1097/HTR.0000000000000090 PMC5594410

[8] Ponsford JL and Spitz G. Stability of employment over the first 3 years following traumatic brain injury. J Head Trauma Rehabil. 2015; 30(3):E1–11. doi: 10.1097/HTR.0000000000000033 24816157 10.1097/HTR.0000000000000033

[9] Ghosh M and Pearse DD. Cyclic amp-specific pdes: A promising therapeutic target for cns repair. Transl Neurosci. 2010; 1(2):101–105.

[10] Li L-X, Cheng Y-F, Lin H-B, Wang C, Xu J-P, and Zhang H-T. Prevention of cerebral ischemia-induced memory deficits by inhibition of phosphodiesterase-4 in rats. Metab Brain Dis. 2011; 26(1):37–47. doi: 10.1007/s11011-011-9235-0 21327879 10.1007/s11011-011-9235-0

[11] Sommer N, Loschmann PA, Northoff GH, Weller M, Steinbrecher A, Steinbach JP, et al The antidepressant rolipram suppresses cytokine production and prevents autoimmune encephalomyelitis. Nat Med. 1995; 1(3):244–8. 7585041 10.1038/nm0395-244

[12] Atkins CM, Oliva AA Jr., Alonso OF, Pearse DD, Bramlett HM, and Dietrich WD. Modulation of the camp signaling pathway after traumatic brain injury. Exp Neurol. 2007; 208(1):145–58. doi: 10.1016/j.expneurol.2007.08.011 17916353 10.1016/j.expneurol.2007.08.011 PMC2141537

[13] Schaal SM, Garg MS, Ghosh M, Lovera L, Lopez M, Patel M, et al The therapeutic profile of rolipram, pde target and mechanism of action as a neuroprotectant following spinal cord injury. PLoS One. 2012; 7(9):e43634 doi: 10.1371/journal.pone.0043634 23028463 10.1371/journal.pone.0043634 PMC3446989

[14] Houslay MD. Underpinning compartmentalised camp signalling through targeted camp breakdown. Trends Biochem Sci. 2010; 35(2):91–100. doi: 10.1016/j.tibs.2009.09.007 19864144 10.1016/j.tibs.2009.09.007

[15] Ghosh M, Garcia-Castillo D, Aguirre V, Golshani R, Atkins CM, Bramlett HM, et al Proinflammatory cytokine regulation of cyclic amp-phosphodiesterase 4 signaling in microglia in vitro and following cns injury. Glia. 2012; 60(12):1839–59. doi: 10.1002/glia.22401 22865690 10.1002/glia.22401 PMC4383287

[16] Hätinen S, Sairanen M, Sirviö J, and Jolkkonen J. Improved sensorimotor function by rolipram following focal cerebral ischemia in rats. Restor Neurol Neurosci. 2008; 26(6):493–499. 19096137

[17] Jin SL, Ding SL, and Lin SC. Phosphodiesterase 4 and its inhibitors in inflammatory diseases. Chang Gung Med J. 2012; 35(3):197–210. 22735051 10.4103/2319-4170.106152

[18] Flora G, Joseph G, Patel S, Singh A, Bleicher D, Barakat DJ, et al Combining neurotrophin-transduced schwann cells and rolipram to promote functional recovery from subacute spinal cord injury. Cell Transplant. 2013; 22(12):2203–17. doi: 10.3727/096368912X658872 23146351 10.3727/096368912X658872

[19] Houslay MD and Adams DR. Putting the lid on phosphodiesterase 4. Nat Biotechnol. 2010; 28(1):38–40. doi: 10.1038/nbt0110-38 20062038 10.1038/nbt0110-38

[20] Giembycz MA and Field SK. Roflumilast: First phosphodiesterase 4 inhibitor approved for treatment of copd. Drug Des Devel Ther. 2010; 4:147–158. 20689641 10.2147/dddt.s7667 PMC2915539

[21] Atkins CM, Kang Y, Furones C, Truettner JS, Alonso OF, and Dietrich WD. Postinjury treatment with rolipram increases hemorrhage after traumatic brain injury. J Neurosci Res. 2012; 90(9):1861–71. doi: 10.1002/jnr.23069 22535545 10.1002/jnr.23069 PMC3418599

[22] Atkins CM, Cepero ML, Kang Y, Liebl DJ, and Dietrich WD. Effects of early rolipram treatment on histopathological outcome after controlled cortical impact injury in mice. Neurosci Lett. 2013; 532:1–6. doi: 10.1016/j.neulet.2012.10.019 23103712 10.1016/j.neulet.2012.10.019 PMC3527646

[23] Robichaud A, Savoie C, Stamatiou PB, Tattersall FD, and Chan CC. Pde4 inhibitors induce emesis in ferrets via a noradrenergic pathway. Neuropharmacol. 2001; 40(2):262–9. 10.1016/s0028-3908(00)00142-8 11114405

[24] Robichaud A, Stamatiou PB, Jin SL, Lachance N, MacDonald D, Laliberte F, et al Deletion of phosphodiesterase 4d in mice shortens alpha(2)-adrenoceptor-mediated anesthesia, a behavioral correlate of emesis. J Clin Invest. 2002; 110(7):1045–52. doi: 10.1172/JCI15506 12370283 10.1172/JCI15506 PMC151147

[25] Jin SL, Lan L, Zoudilova M, and Conti M. Specific role of phosphodiesterase 4b in lipopolysaccharide-induced signaling in mouse macrophages. J Immunol. 2005; 175(3):1523–31. 16034090 10.4049/jimmunol.175.3.1523

[26] Ariga M, Neitzert B, Nakae S, Mottin G, Bertrand C, Pruniaux MP, et al Nonredundant function of phosphodiesterases 4d and 4b in neutrophil recruitment to the site of inflammation. J Immunol. 2004; 173(12):7531–8. 15585880 10.4049/jimmunol.173.12.7531

[27] Soares HD, Hicks RR, Smith D, and McIntosh TK. Inflammatory leukocytic recruitment and diffuse neuronal degeneration are separate pathological processes resulting from traumatic brain injury. J Neurosci. 1995; 15(12):8223–33. 8613756 10.1523/JNEUROSCI.15-12-08223.1995 PMC6577921

[28] Keeling KL, Hicks RR, Mahesh J, Billings BB, and Kotwal GJ. Local neutrophil influx following lateral fluid-percussion brain injury in rats is associated with accumulation of complement activation fragments of the third component (c3) of the complement system. J Neuroimmunol. 2000; 105(1):20–30. 10713360 10.1016/s0165-5728(00)00183-1

[29] Gyoneva S and Ransohoff RM. Inflammatory reaction after traumatic brain injury: Therapeutic potential of targeting cell–cell communication by chemokines. Trends Pharmacol Sci. 2015; 36(7):471–480. doi: 10.1016/j.tips.2015.04.003 25979813 10.1016/j.tips.2015.04.003 PMC4485943

[30] Biagas KV, Uhl MW, Schiding JK, Nemoto EM, and Kochanek PM. Assessment of posttraumatic polymorphonuclear leukocyte accumulation in rat brain using tissue myeloperoxidase assay and vinblastine treatment. J Neurotrauma. 1992; 9(4):363–71. doi: 10.1089/neu.1992.9.363 1337917 10.1089/neu.1992.9.363

[31] Schoettle RJ, Kochanek PM, Magargee MJ, Uhl MW, and Nemoto EM. Early polymorphonuclear leukocyte accumulation correlates with the development of posttraumatic cerebral edema in rats. J Neurotrauma. 1990; 7(4):207–17. doi: 10.1089/neu.1990.7.207 2127947 10.1089/neu.1990.7.207

[32] Utagawa A, Bramlett HM, Daniels L, Lotocki G, Dekaban GA, Weaver LC, et al Transient blockage of the cd11d/cd18 integrin reduces contusion volume and macrophage infiltration after traumatic brain injury in rats. Brain Res. 2008; 1207:155–63. doi: 10.1016/j.brainres.2008.02.057 18374312 10.1016/j.brainres.2008.02.057 PMC2435262

[33] Shultz SR, Bao F, Weaver LC, Cain DP, and Brown A. Treatment with an anti-cd11d integrin antibody reduces neuroinflammation and improves outcome in a rat model of repeated concussion. J Neuroinflammation. 2013; 10(1):793. 10.1186/1742-2094-10-26 PMC3598378 23414334

[34] Erdely A, Kepka-Lenhart D, Clark M, Zeidler-Erdely P, Poljakovic M, Calhoun WJ, et al Inhibition of phosphodiesterase 4 amplifies cytokine-dependent induction of arginase in macrophages. Am J Physiol Lung Cell Mol Physiol. 2006; 290(3):L534–9. doi: 10.1152/ajplung.00326.2005 16257997 10.1152/ajplung.00326.2005

[35] Gray MJ, Poljakovic M, Kepka-Lenhart D, and Morris SM Jr. Induction of arginase i transcription by il-4 requires a composite DNA response element for stat6 and c/ebpbeta. Gene. 2005; 353(1):98–106. doi: 10.1016/j.gene.2005.04.004 15922518 10.1016/j.gene.2005.04.004

[36] Sheldon KE, Shandilya H, Kepka-Lenhart D, Poljakovic M, Ghosh A, and Morris SM. Shaping the murine macrophage phenotype: Il-4 and camp synergistically activate the arginase i promoter. J Immunol. 2013; 191(5):2290–2298. doi: 10.4049/jimmunol.1202102 23913966 10.4049/jimmunol.1202102 PMC3829606

[37] Wei LH, Jacobs AT, Morris SM Jr., and Ignarro LJ. Il-4 and il-13 upregulate arginase i expression by camp and jak/stat6 pathways in vascular smooth muscle cells. Am J Physiol Cell Physiol. 2000; 279(1):C248–56. 10898736 10.1152/ajpcell.2000.279.1.C248

[38] Ghosh M, Xu Y, and Pearse DD. Cyclic amp is a key regulator of m1 to m2a phenotypic conversion of microglia in the presence of th2 cytokines. J Neuroinflammation. 2016; 13:9 doi: 10.1186/s12974-015-0463-9 26757726 10.1186/s12974-015-0463-9 PMC4711034

[39] Bronte V and Zanovello P. Regulation of immune responses by l-arginine metabolism. Nat Rev Immunol. 2005; 5(8):641–54. doi: 10.1038/nri1668 16056256 10.1038/nri1668

[40] Munder M. Arginase: An emerging key player in the mammalian immune system. Br J Pharmacol. 2009; 158(3):638–51. doi: 10.1111/j.1476-5381.2009.00291.x 19764983 10.1111/j.1476-5381.2009.00291.x PMC2765586

[41] Rath M, Muller I, Kropf P, Closs EI, and Munder M. Metabolism via arginase or nitric oxide synthase: Two competing arginine pathways in macrophages. Front Immunol. 2014; 5:532 doi: 10.3389/fimmu.2014.00532 25386178 10.3389/fimmu.2014.00532 PMC4209874

[42] Shepherd MC, Baillie GS, Stirling DI, and Houslay MD. Remodelling of the pde4 camp phosphodiesterase isoform profile upon monocyte-macrophage differentiation of human u937 cells. Br J Pharmacol. 2004; 142(2):339–51. doi: 10.1038/sj.bjp.0705770 15066910 10.1038/sj.bjp.0705770 PMC1574950

[43] Wang P, Wu P, Ohleth KM, Egan RW, and Billah MM. Phosphodiesterase 4b2 is the predominant phosphodiesterase species and undergoes differential regulation of gene expression in human monocytes and neutrophils. Mol Pharmacol. 1999; 56(1):170–4. 10385698 10.1124/mol.56.1.170

[44] Reyes-Irisarri E, Perez-Torres S, Miro X, Martinez E, Puigdomenech P, Palacios JM, et al Differential distribution of pde4b splice variant mrnas in rat brain and the effects of systemic administration of lps in their expression. Synapse. 2008; 62(1):74–9. doi: 10.1002/syn.20459 17960764 10.1002/syn.20459

[45] Oliva AA Jr., Kang Y, Furones C, Alonso OF, Bruno O, Dietrich WD, et al Phosphodiesterase isoform-specific expression induced by traumatic brain injury. J Neurochem. 2012; 123(6):1019–29. doi: 10.1111/jnc.12049 23057870 10.1111/jnc.12049 PMC3514616

[46] Reyes-Irisarri E, Sanchez AJ, Garcia-Merino JA, and Mengod G. Selective induction of camp phosphodiesterase pde4b2 expression in experimental autoimmune encephalomyelitis. J Neuropathol Exp Neurol. 2007; 66(10):923–31. doi: 10.1097/nen.0b013e3181567c31 17917586 10.1097/nen.0b013e3181567c31

[47] Zhang C, Xu Y, Zhang HT, Gurney ME, and O'Donnell JM. Comparison of the pharmacological profiles of selective pde4b and pde4d inhibitors in the central nervous system. Sci Rep. 2017; 7:40115 doi: 10.1038/srep40115 28054669 10.1038/srep40115 PMC5215650

[48] Naganuma K, Omura A, Maekawara N, Saitoh M, Ohkawa N, Kubota T, et al Discovery of selective pde4b inhibitors. Bioorg Med Chem Lett. 2009; 19(12):3174–6. doi: 10.1016/j.bmcl.2009.04.121 19447034 10.1016/j.bmcl.2009.04.121

[49] Fox D 3rd, Burgin AB, and Gurney ME. Structural basis for the design of selective phosphodiesterase 4b inhibitors. Cell Signal. 2014; 26(3):657–63. doi: 10.1016/j.cellsig.2013.12.003 24361374 10.1016/j.cellsig.2013.12.003 PMC4057648

[50] Titus DJ, Wilson NM, Freund JE, Carballosa MM, Sikah KE, Furones C, et al Chronic cognitive dysfunction after traumatic brain injury is improved with a phosphodiesterase 4b inhibitor. J Neurosci. 2016; 36(27):7095–108. doi: 10.1523/JNEUROSCI.3212-15.2016 27383587 10.1523/JNEUROSCI.3212-15.2016 PMC4938858

[51] Hagen TJ, Mo X, Burgin AB, Fox D 3rd, Zhang Z, and Gurney ME. Discovery of triazines as selective pde4b versus pde4d inhibitors. Bioorg Med Chem Lett. 2014; 24(16):4031–4. doi: 10.1016/j.bmcl.2014.06.002 24998378 10.1016/j.bmcl.2014.06.002 PMC4142572

[52] Eigler A, Siegmund B, Emmerich U, Baumann KH, Hartmann G, and Endres S. Anti-inflammatory activities of camp-elevating agents: Enhancement of il-10 synthesis and concurrent suppression of tnf production. J Leukoc Biol. 1998; 63(1):101–7. 9469479 10.1002/jlb.63.1.101

[53] Blaya MO, Bramlett HM, Naidoo J, Pieper AA, and Dietrich WD. Neuroprotective efficacy of a proneurogenic compound after traumatic brain injury. J Neurotrauma. 2014; 31(5):476–86. doi: 10.1089/neu.2013.3135 24070637 10.1089/neu.2013.3135 PMC3934600

[54] Schaar KL, Brenneman MM, and Savitz SI. Functional assessments in the rodent stroke model. Exp Transl Stroke Med. 2010; 2(13). 10.1186/2040-7378-2-13 PMC2915950 20642841

[55] Rudy JW and O'Reilly RC. Contextual fear conditioning, conjunctive representations, pattern completion, and the hippocampus. Behav Neurosci. 1999; 113(5):867–80. 10571471 10.1037//0735-7044.113.5.867

[56] Titus DJ, Furones C, Kang Y, and Atkins CM. Age-dependent alterations in camp signaling contribute to synaptic plasticity deficits following traumatic brain injury. Neurosci. 2013; 231:182–94. 10.1016/j.neuroscience.2012.12.002 PMC3558555 23238576

[57] Gotoh S, Itoh M, Fujii Y, Arai S, and Sendo F. Enhancement of the expression of a rat neutrophil-specific cell surface antigen by activation with phorbol myristate acetate and concanavalin a. J Immunol. 1986; 137(2):643–50. 2424975

[58] Vander Top EA, Perry GA, and Gentry-Nielsen MJ. A novel flow cytometric assay for measurement of in vivo pulmonary neutrophil phagocytosis. BMC Microbiology. 2006; 6(1):61. 16836747 10.1186/1471-2180-6-61 PMC1533832

[59] Maas AI, Stocchetti N, and Bullock R. Moderate and severe traumatic brain injury in adults. Lancet Neurol. 2008; 7(8):728–41. doi: 10.1016/S1474-4422(08)70164-9 18635021 10.1016/S1474-4422(08)70164-9

[60] Utagawa A, Truettner JS, Dietrich WD, and Bramlett HM. Systemic inflammation exacerbates behavioral and histopathological consequences of isolated traumatic brain injury in rats. Exp Neurol. 2008; 211(1):283–91. doi: 10.1016/j.expneurol.2008.02.001 18355811 10.1016/j.expneurol.2008.02.001 PMC2435258

[61] Schallert T, Fleming SM, Leasure JL, Tillerson JL, and Bland ST. Cns plasticity and assessment of forelimb sensorimotor outcome in unilateral rat models of stroke, cortical ablation, parkinsonism and spinal cord injury. Neuropharm. 2000; 39(5):777–787. 10.1016/s0028-3908(00)00005-8 10699444

[62] Baskin YK, Dietrich WD, and Green EJ. Two effective behavioral tasks for evaluating sensorimotor dysfunction following traumatic brain injury in mice. J Neurosci Methods. 2003; 129(1):87–93. 12951236 10.1016/s0165-0270(03)00212-7

[63] Dash PK, Moore AN, Kobori N, and Runyan JD. Molecular activity underlying working memory. Learn Mem. 2007; 14(8):554–63. doi: 10.1101/lm.558707 17690339 10.1101/lm.558707

[64] Titus DJ, Sakurai A, Kang Y, Furones C, Jergova S, Santos R, et al Phosphodiesterase inhibition rescues chronic cognitive deficits induced by traumatic brain injury. J Neurosci. 2013; 33(12):5216–26. doi: 10.1523/JNEUROSCI.5133-12.2013 23516287 10.1523/JNEUROSCI.5133-12.2013 PMC3655415

[65] Smith DH, Chen XH, Pierce JE, Wolf JA, Trojanowski JQ, Graham DI, et al Progressive atrophy and neuron death for one year following brain trauma in the rat. J Neurotrauma. 1997; 14(10):715–27. doi: 10.1089/neu.1997.14.715 9383090 10.1089/neu.1997.14.715

[66] Bramlett HM and Dietrich WD. Quantitative structural changes in white and gray matter 1 year following traumatic brain injury in rats. Acta Neuropathol. 2002; 103(6):607–14. doi: 10.1007/s00401-001-0510-8 12012093 10.1007/s00401-001-0510-8

[67] Kabadi SV, Hilton GD, Stoica BA, Zapple DN, and Faden AI. Fluid-percussion-induced traumatic brain injury model in rats. Nat Protoc. 2010; 5(9):1552–63. doi: 10.1038/nprot.2010.112 20725070 10.1038/nprot.2010.112 PMC3753081

[68] Truettner JS, Suzuki T, and Dietrich WD. The effect of therapeutic hypothermia on the expression of inflammatory response genes following moderate traumatic brain injury in the rat. Brain Res Mol Brain Res. 2005; 138(2):124–34. doi: 10.1016/j.molbrainres.2005.04.006 15922484 10.1016/j.molbrainres.2005.04.006

[69] Knoblach SM and Faden AI. Interleukin-10 improves outcome and alters proinflammatory cytokine expression after experimental traumatic brain injury. Exp Neurol. 1998; 153(1):143–51. doi: 10.1006/exnr.1998.6877 9743576 10.1006/exnr.1998.6877

[70] Kumar A and Loane DJ. Neuroinflammation after traumatic brain injury: Opportunities for therapeutic intervention. Brain Behav Immun. 2012; 26(8):1191–201. doi: 10.1016/j.bbi.2012.06.008 22728326 10.1016/j.bbi.2012.06.008

[71] Lotocki G, de Rivero Vaccari JP, Perez ER, Sanchez-Molano J, Furones-Alonso O, Bramlett HM, et al Alterations in blood-brain barrier permeability to large and small molecules and leukocyte accumulation after traumatic brain injury: Effects of post-traumatic hypothermia. J Neurotrauma. 2009; 26(7):1123–34. doi: 10.1089/neu.2008.0802 19558276 10.1089/neu.2008.0802 PMC2848945

[72] Blackman BE, Horner K, Heidmann J, Wang D, Richter W, Rich TC, et al Pde4d and pde4b function in distinct subcellular compartments in mouse embryonic fibroblasts. J Biol Chem. 2011; 286(14):12590–601. doi: 10.1074/jbc.M110.203604 21288894 10.1074/jbc.M110.203604 PMC3069460

[73] Millar JK, Pickard BS, Mackie S, James R, Christie S, Buchanan SR, et al Disc1 and pde4b are interacting genetic factors in schizophrenia that regulate camp signaling. Science. 2005; 310(5751):1187–91. doi: 10.1126/science.1112915 16293762 10.1126/science.1112915

[74] Wilson NM, Titus DJ, Oliva AA Jr., Furones C, and Atkins CM. Traumatic brain injury upregulates phosphodiesterase expression in the hippocampus. Front Syst Neurosci. 2016; 10:5 doi: 10.3389/fnsys.2016.00005 26903822 10.3389/fnsys.2016.00005 PMC4742790

[75] Ma D, Wu P, Egan RW, Billah MM, and Wang P. Phosphodiesterase 4b gene transcription is activated by lipopolysaccharide and inhibited by interleukin-10 in human monocytes. Mol Pharmacol. 1999; 55(1):50–7. 9882697 10.1124/mol.55.1.50

[76] Zhu HT, Bian C, Yuan JC, Chu WH, Xiang X, Chen F, et al Curcumin attenuates acute inflammatory injury by inhibiting the tlr4/myd88/nf-kappab signaling pathway in experimental traumatic brain injury. J Neuroinflammation. 2014; 11:59 doi: 10.1186/1742-2094-11-59 24669820 10.1186/1742-2094-11-59 PMC3986937

[77] Ye Y, Xu H, Zhang X, Li Z, Jia Y, He X, et al Association between toll-like receptor 4 expression and neural stem cell proliferation in the hippocampus following traumatic brain injury in mice. Int J Mol Sci. 2014; 15(7):12651–64. doi: 10.3390/ijms150712651 25036030 10.3390/ijms150712651 PMC4139865

[78] Laird MD, Shields JS, Sukumari-Ramesh S, Kimbler DE, Fessler RD, Shakir B, et al High mobility group box protein-1 promotes cerebral edema after traumatic brain injury via activation of toll-like receptor 4. Glia. 2014; 62(1):26–38. doi: 10.1002/glia.22581 24166800 10.1002/glia.22581 PMC4503251

[79] Ahmad A, Crupi R, Campolo M, Genovese T, Esposito E, and Cuzzocrea S. Absence of tlr4 reduces neurovascular unit and secondary inflammatory process after traumatic brain injury in mice. PLoS One. 2013; 8(3):e57208 doi: 10.1371/journal.pone.0057208 23555560 10.1371/journal.pone.0057208 PMC3610903

[80] Lynn WA, Raetz CR, Qureshi N, and Golenbock DT. Lipopolysaccharide-induced stimulation of cd11b/cd18 expression on neutrophils. Evidence of specific receptor-based response and inhibition by lipid a-based antagonists. J Immunol. 1991; 147(9):3072–9. 1717586

[81] Detmers PA, Zhou D, and Powell DE. Different signaling pathways for cd18-mediated adhesion and fc-mediated phagocytosis. Response of neutrophils to lps. J Immunol. 1994; 153(5):2137–45. 7519640

[82] Zhou X, Gao XP, Fan J, Liu Q, Anwar KN, Frey RS, et al Lps activation of toll-like receptor 4 signals cd11b/cd18 expression in neutrophils. Am J Physiol Lung Cell Mol Physiol. 2005; 288(4):L655–62. doi: 10.1152/ajplung.00327.2004 15563689 10.1152/ajplung.00327.2004

[83] Corraliza IM, Soler G, Eichmann K, and Modolell M. Arginase induction by suppressors of nitric oxide synthesis (il-4, il-10 and pge2) in murine bone-marrow-derived macrophages. Biochem Biophys Res Commun. 1995; 206. 10.1006/bbrc.1995.1094 7530004

[84] Truettner JS, Bramlett HM, and Dietrich WD. Posttraumatic therapeutic hypothermia alters microglial and macrophage polarization toward a beneficial phenotype. J Cereb Blood Flow Metab. 2016. 10.1177/0271678X16680003 PMC5536802 27864465

[85] Kumar A, Alvarez-Croda DM, Stoica BA, Faden AI, and Loane DJ. Microglial/macrophage polarization dynamics following traumatic brain injury. J Neurotrauma. 2016; 33(19):1732–1750. doi: 10.1089/neu.2015.4268 26486881 10.1089/neu.2015.4268 PMC5065034

[86] Gensel JC and Zhang B. Macrophage activation and its role in repair and pathology after spinal cord injury. Brain Research. 2015; 1619:1–11. doi: 10.1016/j.brainres.2014.12.045 25578260 10.1016/j.brainres.2014.12.045

[87] Ferrante CJ and Leibovich SJ. Regulation of macrophage polarization and wound healing. Adv Wound Care. 2012; 1(1):10–16. 10.1089/wound.2011.0307 PMC3623587 24527272

[88] Roszer T. Understanding the mysterious m2 macrophage through activation markers and effector mechanisms. Mediators Inflamm. 2015. 10.1155/2015/816460 PMC4452191 26089604

[89] Chang CI, Zoghi B, Liao JC, and Kuo L. The involvement of tyrosine kinases, cyclic amp/protein kinase a, and p38 mitogen-activated protein kinase in il-13-mediated arginase i induction in macrophages: Its implications in il-13-inhibited nitric oxide production. J Immunol. 2000; 165(4):2134–41. 10925299 10.4049/jimmunol.165.4.2134

[90] Siegmund B, Eigler A, Moeller J, Greten TF, Hartmann G, and Endres S. Suppression of tumor necrosis factor-alpha production by interleukin-10 is enhanced by camp-elevating agents. Eur J Pharmacol. 1997; 321(2):231–9. 9063693 10.1016/s0014-2999(96)00947-8

[91] Gordon S. Alternative activation of macrophages. Nat Rev Immunol. 2003; 3(1):23–35. doi: 10.1038/nri978 12511873 10.1038/nri978

[92] Cherry JD, Olschowka JA, and O’Banion MK. Neuroinflammation and m2 microglia: The good, the bad, and the inflamed. J Neuroinflammation. 2014; 11(1):98. 24889886 10.1186/1742-2094-11-98 PMC4060849

[93] Pauleau AL, Rutschman R, Lang R, Pernis A, Watowich SS, and Murray PJ. Enhancer-mediated control of macrophage-specific arginase i expression. J Immunol. 2004; 172(12):7565–73. 15187136 10.4049/jimmunol.172.12.7565

[94] Godoy DA, Rubiano A, Rabinstein AA, Bullock R, and Sahuquillo J. Moderate traumatic brain injury: The grey zone of neurotrauma. Neurocrit Care. 2016; 25(2):306–19. doi: 10.1007/s12028-016-0253-y 26927279 10.1007/s12028-016-0253-y

[95] Munch E, Horn P, Schurer L, Piepgras A, Paul T, and Schmiedek P. Management of severe traumatic brain injury by decompressive craniectomy. Neurosurgery. 2000; 47(2):315–22. 10942004 10.1097/00006123-200008000-00009

[96] Stiver SI. Complications of decompressive craniectomy for traumatic brain injury. Neurosurg Focus. 2009; 26(6):E7 doi: 10.3171/2009.4.FOCUS0965 19485720 10.3171/2009.4.FOCUS0965

[97] de Rivero Vaccari JP, Lotocki G, Alonso OF, Bramlett HM, Dietrich WD, and Keane RW. Therapeutic neutralization of the nlrp1 inflammasome reduces the innate immune response and improves histopathology after traumatic brain injury. J Cereb Blood Flow Metab. 2009; 29(7):1251–61. doi: 10.1038/jcbfm.2009.46 19401709 10.1038/jcbfm.2009.46 PMC2846547

[98] Fukuda K, Tanno H, Okimura Y, Nakamura M, and Yamaura A. The blood-brain barrier disruption to circulating proteins in the early period after fluid percussion brain injury in rats. J Neurotrauma. 1995; 12(3):315–24. doi: 10.1089/neu.1995.12.315 7473806 10.1089/neu.1995.12.315

[99] Jiang JY, Lyeth BG, Kapasi MZ, Jenkins LW, and Povlishock JT. Moderate hypothermia reduces blood-brain barrier disruption following traumatic brain injury in the rat. Acta Neuropathol. 1992; 84(5):495–500. 1462764 10.1007/BF00304468

[100] Dejana E, Orsenigo F, and Lampugnani MG. The role of adherens junctions and ve-cadherin in the control of vascular permeability. J Cell Sci. 2008; 121(13):2115–22. 18565824 10.1242/jcs.017897

[101] Cullere X, Shaw SK, Andersson L, Hirahashi J, Luscinskas FW, and Mayadas TN. Regulation of vascular endothelial barrier function by epac, a camp-activated exchange factor for rap gtpase. Blood. 2005; 105(5):1950–5. doi: 10.1182/blood-2004-05-1987 15374886 10.1182/blood-2004-05-1987

[102] Moy AB, Bodmer JE, Blackwell K, Shasby S, and Shasby DM. Camp protects endothelial barrier function independent of inhibiting mlc20-dependent tension development. Am J Physiol. 1998; 274(6):1024–9. 10.1152/ajplung.1998.274.6.L1024 9609742

[103] Pati S, Khakoo AY, Zhao J, Jimenez F, Gerber MH, Harting M, et al Human mesenchymal stem cells inhibit vascular permeability by modulating vascular endothelial cadherin/β-catenin signaling. Stem Cells Dev. 2011; 20(1):89–101. doi: 10.1089/scd.2010.0013 20446815 10.1089/scd.2010.0013 PMC3128758

[104] Abel T and Lattal KM. Molecular mechanisms of memory acquisition, consolidation and retrieval. Curr Opin Neurobiol. 2001; 11(2):180–7. 11301237 10.1016/s0959-4388(00)00194-x

[105] McKenzie S and Eichenbaum H. Consolidation and reconsolidation: Two lives of memories? Neuron. 2011; 71(2):224–33. doi: 10.1016/j.neuron.2011.06.037 21791282 10.1016/j.neuron.2011.06.037 PMC3145971

[106] Smith DH, Chen XH, Pierce JE, Wolf JA, Trojanowski JQ, and Graham DI. Progressive atrophy and neuron death for one year following brain trauma in the rat. J Neurotrauma. 1997; 14(10):715–27. doi: 10.1089/neu.1997.14.715 9383090 10.1089/neu.1997.14.715

[107] Winston CN, Chellappa D, Wilkins T, Barton DJ, Washington PM, Loane DJ, et al Controlled cortical impact results in an extensive loss of dendritic spines that is not mediated by injury-induced amyloid-beta accumulation. J Neurotrauma. 2013; 30(23):1966–72. doi: 10.1089/neu.2013.2960 23879560 10.1089/neu.2013.2960 PMC3837436

[108] Gao X, Deng P, Xu ZC, and Chen J. Moderate traumatic brain injury causes acute dendritic and synaptic degeneration in the hippocampal dentate gyrus. PLoS One. 2011; 6(9):e24566 doi: 10.1371/journal.pone.0024566 21931758 10.1371/journal.pone.0024566 PMC3172233

[109] Scheff SW, Price DA, Hicks RR, Baldwin SA, Robinson S, and Brackney C. Synaptogenesis in the hippocampal ca1 field following traumatic brain injury. J Neurotrauma. 2005; 22(7):719–32. doi: 10.1089/neu.2005.22.719 16004576 10.1089/neu.2005.22.719

[110] Block F, Tondar A, Schmidt W, and Schwarz M. Delayed treatment with rolipram protects against neuronal damage following global ischemia in rats. Neuroreport. 1997; 8(17):3829–32. 9427378 10.1097/00001756-199712010-00033

[111] Hicks R, Soares H, Smith D, and McIntosh T. Temporal and spatial characterization of neuronal injury following lateral fluid-percussion brain injury in the rat. Acta Neuropathol. 1996; 91(3):236–46. 8834535 10.1007/s004010050421

[112] Sato M, Chang E, Igarashi T, and Noble LJ. Neuronal injury and loss after traumatic brain injury: Time course and regional variability. Brain Res. 2001; 917(1):45–54. 11602228 10.1016/s0006-8993(01)02905-5

[113] Michael LF, Asahara H, Shulman AI, Kraus WL, and Montminy M. The phosphorylation status of a cyclic amp-responsive activator is modulated via a chromatin-dependent mechanism. Mol Cell Biol. 2000; 20(5):1596–603. 10669737 10.1128/mcb.20.5.1596-1603.2000 PMC85343

[114] Meller R, Minami M, Cameron JA, Impey S, Chen D, Lan JQ, et al Creb-mediated bcl-2 protein expression after ischemic preconditioning. J Cereb Blood Flow Metab. 2005; 25(2):234–46. doi: 10.1038/sj.jcbfm.9600024 15647742 10.1038/sj.jcbfm.9600024

[115] Raghupathi R, Fernandez SC, Murai H, Trusko SP, Scott RW, Nishioka WK, et al Bcl-2 overexpression attenuates cortical cell loss after traumatic brain injury in transgenic mice. J Cereb Blood Flow Metab. 1998; 18(11):1259–1269. doi: 10.1097/00004647-199811000-00013 9809516 10.1097/00004647-199811000-00013

[116] Nakamura M, Raghupathi R, Merry DE, Scherbel U, Saatman KE, and McIntosh TK. Overexpression of bcl-2 is neuroprotective after experimental brain injury in transgenic mice. J Comp Neurol. 1999; 412(4):681–92. 10464363 10.1002/(sici)1096-9861(19991004)412:4<681::aid-cne9>3.0.co;2-f

[117] Lee J, Kim CH, Simon DK, Aminova LR, Andreyev AY, Kushnareva YE, et al Mitochondrial cyclic amp response element-binding protein (creb) mediates mitochondrial gene expression and neuronal survival. J Biol Chem. 2005; 280(49):40398–401. doi: 10.1074/jbc.C500140200 16207717 10.1074/jbc.C500140200 PMC2612541

[118] James R, Adams RR, Christie S, Buchanan SR, Porteous DJ, and Millar JK. Disrupted in schizophrenia 1 (disc1) is a multicompartmentalized protein that predominantly localizes to mitochondria. Mol Cell Neurosci. 2004; 26(1):112–122. doi: 10.1016/j.mcn.2004.01.013 15121183 10.1016/j.mcn.2004.01.013

[119] Vitarbo EA, Chatzipanteli K, Kinoshita K, Truettner JS, Alonso OF, and Dietrich WD. Tumor necrosis factor α expression and protein levels after fluid percussion injury in rats: The effect of injury severity and brain temperature. Neurosurgery. 2004; 55(2):416–425. 15271250 10.1227/01.neu.0000130036.52521.2c

[120] Kinoshita K, Chatzipanteli i K, Vitarbo E, Truettner JS, Alonso OF, and Dietrich WD. Interleukin-1beta messenger ribonucleic acid and protein levels after fluid-percussion brain injury in rats: Importance of injury severity and brain temperature. Neurosurgery. 2002; 51(1):195–203. 10.1097/00006123-200207000-00027 12182417

[121] Lotocki G, Alonso OF, Dietrich WD, and Keane RW. Tumor necrosis factor receptor 1 and its signaling intermediates are recruited to lipid rafts in the traumatized brain. J Neurosci. 2004; 24(49):11010–6. doi: 10.1523/JNEUROSCI.3823-04.2004 15590916 10.1523/JNEUROSCI.3823-04.2004 PMC6730274

[122] Sugerman DE, Xu L, Pearson WS, and Faul M. Patients with severe traumatic brain injury transferred to a level i or ii trauma center: United states, 2007 to 2009. J Trauma Acute Care Surg. 2012; 73(6):1491–9. doi: 10.1097/TA.0b013e3182782675 23188242 10.1097/TA.0b013e3182782675

[123] Hartl R, Gerber LM, Iacono L, Ni Q, Lyons K, and Ghajar J. Direct transport within an organized state trauma system reduces mortality in patients with severe traumatic brain injury. J Trauma. 2006; 60(6):1250–6. doi: 10.1097/01.ta.0000203717.57821.8d 16766968 10.1097/01.ta.0000203717.57821.8d

[124] Li YF, Cheng YF, Huang Y, Conti M, Wilson SP, O'Donnell JM, et al Phosphodiesterase-4d knock-out and rna interference-mediated knock-down enhance memory and increase hippocampal neurogenesis via increased camp signaling. J Neurosci. 2011; 31(1):172–83. doi: 10.1523/JNEUROSCI.5236-10.2011 21209202 10.1523/JNEUROSCI.5236-10.2011 PMC3079568

[125] Havekes R, Park AJ, Tolentino RE, Bruinenberg VM, Tudor JC, Lee Y, et al Compartmentalized pde4a5 signaling impairs hippocampal synaptic plasticity and long-term memory. J Neurosci. 2016; 36(34):8936–46. doi: 10.1523/JNEUROSCI.0248-16.2016 27559174 10.1523/JNEUROSCI.0248-16.2016 PMC4995304

[126] Sheppard H and Tsien WH. Alterations in the hydrolytic activity, inhibitor sensitivity and molecular size of the rat erythrocyte cyclic amp phosphodiesterase by calcium and hypertonic sodium chloride. J Cyclic Nucleotide Res. 1975; 1(4):237–42. 177464

[127] Schwabe U, Miyake M, Ohga Y, and Daly JW. 4-(3-cyclopentyloxy-4-methoxyphenyl)-2-pyrrolidone (zk 62711): A potent inhibitor of adenosine cyclic 3',5'-monophosphate phosphodiesterases in homogenates and tissue slices from rat brain. Mol Pharmacol. 1976; 12(6):900–10. 187926

[128] Mullard A. 2011 fda drug approvals. Nat Rev Drug Discov. 2012; 11(2):91–94. doi: 10.1038/nrd3657 22293555 10.1038/nrd3657

[129] FitzGerald O. Spondyloarthropathies: Apremilast: Welcome advance in treatment of psoriatic arthritis. Nat Rev Rheumatol. 2014; 10(7):385–386. doi: 10.1038/nrrheum.2014.77 24846499 10.1038/nrrheum.2014.77

